# *In silico* genomic analysis of the potential probiotic *Lactiplantibacillus pentosus* CF2-10N reveals promising beneficial effects with health promoting properties

**DOI:** 10.3389/fmicb.2022.989824

**Published:** 2022-11-03

**Authors:** Hikmate Abriouel, Julia Manetsberger, Natacha Caballero Gómez, Nabil Benomar

**Affiliations:** Área de Microbiología, Departamento de Ciencias de la Salud, Facultad de Ciencias Experimentales, Universidad de Jaén, Jaén, Spain

**Keywords:** Aloreña table olives, *Lactiplantibacillus pentosus*, probiotics, *in silico* analysis, safety, functional properties

## Abstract

*Lactiplantibacillus pentosus* CF2-10 N, isolated from brines of naturally fermented Aloreña green table olives, exhibited high probiotic potential. High throughput sequencing and annotation of genome sequences underline the potential of *L. pentosus* CF2-10 N as excellent probiotic candidate of vegetable origin. In a previous study we could show the probiotic potential of CF2-10 N *in vitro*, while in this study *in silico* analysis of its genome revealed new insights into its safety and functionality. Our findings highlight the microorganism’s ecological flexibility and adaptability to a broad range of environmental niches, food matrices and the gastrointestinal tract. These features are shared by both phylogenetically very close *L. pentosus* strains (CF2-10 N and MP-10) isolated from the same ecological niche with respect to their genome size (≅ 3.6 Mbp), the presence of plasmids (4–5) and several other properties. Nonetheless, additional and unique features are reported in the present study for *L. pentosus* CF2-10 N. Notably, the safety of *L. pentosus* CF2-10 N was shown by the absence of virulence determinants and the determination of acquired antibiotic resistance genes, i.e., resistome, which is mostly represented by efflux-pump resistance genes responsible for the intrinsic resistance. On the other hand, defense mechanisms of *L. pentosus* CF2-10 N include eight prophage regions and a CRISPR/cas system (CRISPR-I and CRISPR-II) as acquired immune system against mobile elements. Finally, the probiotic potential of this strain was further demonstrated by the presence of genes coding for proteins involved in adhesion, exopolysaccharide biosynthesis, tolerance to low pH and bile salts, immunomodulation, and vitamin and enzyme production. Taken together these results, we propose the use of *L. pentosus* CF2-10 N as a potential and promising probiotic candidate able to colonize several niches and adapt to different lifestyles. The strain can provide attractive functional and probiotic features necessary for its application as starter culture and probiotic.

## Introduction

Probiotics are defined by the Food and Agriculture Organization of the United Nations/World Health Organization (FAO/WHO) “as live microorganisms that, when administered in adequate amounts, confer a health benefit to the host” (Hill et al., 2014). In this regard, probiotic microorganisms are characterized by their diverse origin, taxonomy, fitness, effective dose, host and health benefits depending specifically on the strain employed. Thus, preliminary screening criteria for potential probiotic microorganisms include their capacity to withstand several barriers and challenges (1) *in vitro,* such as stressful environmental conditions; and (2) *in vivo*-notably during their passage through the gastrointestinal tract (acids and bile salts), their capacity to adhere and colonize human epithelial cells and their ability to produce beneficial effects in the host (antimicrobial activity, modulation of the immune system, degradation of toxic components, etc.).

In this sense, the key element for the differentiation of probiotic strains from each other is their specific functionality. Naturally, this has led to a considerable amount of research efforts put into determining the specific probiotic effect(s) of each potential probiotic strain and highlighting their potential targets over recent years (Allain et al., 2018; van de Wijgert and Verwijs, 2019; Yan et al., 2019; Yoha et al., 2022). In other words, a search for unique and attractive functional characteristics is crucial to provide new and helpful information on microorganisms with probiotic potential. This is especially important for those microorganisms that are naturally present in fermented foods, such as for example *Lactiplantibacillus* species.

On the other hand, probiotics as indicated by their name, act as a ‘promoter of life’ supporting in a natural way the improvement of the overall health status of the host organism (Amara and Shibl, 2015). It has further been shown that it is possible to combine several of these strains into multi-strain probiotics (Nayak, 2010), where the strains of this ‘probiotic cocktail’ can work synergistically, thus greatly increasing the overall benefit spectrum for the host (Puvanasundram et al., 2021).

The recently reclassified *Lactiplantibacillus pentosus*, formerly known as *Lactobacillus pentosus* (Zheng et al., 2020), colonizes a large set of environmental niches and therefore exhibits a huge ecological and metabolic adaptability (Anukam et al., 2013; Abriouel et al., 2017; Pérez-Díaz et al., 2021). Due to its genomic diversity and functionality, this species is found in several fermented foods (vegetables, meat, and dairy), plants, animals, vaginal, urogenital and gastrointestinal tract, while also having a large set of biotechnological and probiotic applications (Tofalo et al., 2014; Vaccalluzzo et al., 2020).

*Lactiplantibacillus pentosus* together with *L. plantarum* is an important member of the bacterial community found on the surface of olive fruits and thus represent the predominating bacteria in olive fermentation. Notably, they promote the fermentation process, conservation and extension of shelf life of the product, in addition to their role in organoleptic properties and the production of health promoting molecules such as amino acids, short chain fatty acids (SCFA), antioxidants, exopolysaccharides and vitamins (Caggianiello et al., 2016; Carrasco et al., 2018; Benítez-Cabello et al., 2019; Perpetuini et al., 2020). Furthermore, besides the production of the abovementioned molecules in foods such as olives, these bacteria are also able to produce these substances *in vivo*, i.e., in the gastrointestinal tract thus providing an important probiotic effect (Oguntoyinbo and Narbad, 2015; Saxami et al., 2017; Guantario et al., 2018). Consequently, several fermented foods have been classified as functional foods, as they are carriers of probiotic organisms and/or their molecules. In this regard, the health benefit and functionality of table olives goes beyond just “fermented food” due to their ability to deliver beneficial microbes adhering to the drupe epidermis into the human gastrointestinal tract where they may influence the microbial diversity and functionality (Lavermicocca et al., 2005; Rodríguez-Gómez et al., 2014, 2017).

Among olives, naturally fermented Aloreña green table olives are a promising carrier of probiotics since they are characterized by their diverse microbial community. This is mostly due to the richness of the ecosystem (soil, plant, and brine) and the progressive changes inherent to the production process (Abriouel et al., 2011).

The microbial diversity of Aloreña table olives includes lactic acid bacteria (LAB), mainly *L. pentosus*-yeasts and other contaminant microorganism, with microbial profiles greatly depending on the fermentation conditions (e.g., vat, fermenter or in cold). In this regard, however under vat and fermenter conditions, LAB and yeasts have been determined as the main actors (Abriouel et al., 2011). Among LAB, *L. pentosus* are considered potential probiotics due to their good growth capacity and survival rate under simulated gastro-intestinal conditions (acidic pH of 1.5, up to 4% of bile salts and 5 mM of nitrate), auto-aggregation, co-aggregation with pathogenic bacteria, adhesion to intestinal and vaginal cell lines, biofilm formation, fermentation of several prebiotics and their capacity to ferment lactose among others (Pérez Montoro et al., 2016). In addition, omics approaches were used by our group; including genomics, proteomics and transcriptomics, to determine and confirm the safety and functionality of the probiotic *L. pentosus* isolated from Aloreña table olives (Casado Muñoz et al., 2016; Pérez Montoro et al., 2018a, b; Alonso García et al., 2021, 2023).

Hence, in the present study, we extend the characterization of *L. pentosus* using *in silico* genomic analysis to unveil the genetic basis of the safety and probiotic ability of *L. pentosus* CF2-10 N – one of the most promising potential probiotic strains isolated from Aloreña table olives (Abriouel et al., 2012).

## Materials and methods

### Bacterial strain and growth conditions

*Lactiplantibacillus pentosus* CF2-10 N, originally isolated from naturally fermented Aloreña green table olives (Abriouel et al., 2012), was selected based on its probiotic profile as reported by Pérez Montoro et al. (2016). *Lactiplantibacillus pentosus* CF2-10 N was routinely cultured at 37°C in de Man, Rogosa and Sharpe (MRS) broth or agar (Fluka, Madrid, Spain) under aerobic (atmospheric) conditions for 24–48 h. The strain was kept in 20% glycerol at −80°C for long-term storage.

### DNA extraction, library preparation and genome sequencing

Bacterial cells of *L. pentosus* CF2-10 N were harvested by centrifugation after 18 h incubation at 37°C under aerobic conditions in liquid medium. Total genomic DNA was obtained using the PureGene core kit B, according to the manufacturer’s instructions (Qiagen, Spain). DNA quantification and quality assessment were carried out using a NanoDrop 2000 spectrophotometer (Thermo Scientific), the PicoGreen ds DNA Reagent (Invitrogen) and/or agarose gel electrophoresis (0.8% agarose gel in Tris-borate-EDTA buffer, 90V, 45 min). Bacterial DNA was stored at −20°C until required.

Purified genomic DNA was sheared into 10- to 20-kb fragments using the protocol designed for DNA library preparation using the PacBio RS II System (Pacific Biosciences, Menlo Park, CA, United States). Resulting libraries (22–24 kb) were purified and sequenced using a P6-C4 DNA polymerase (Pacific Biosciences) and single-molecule real-time (SMRT) cells with a 240-min sequence capture protocol and Stage Start to maximize the subread length on the PacBio RS II.

### Genome assembly and annotation

Raw sequence data were filtered (Q20) and a total of 150,292 reads were obtained with a median length of 14,991 bp. The resulting reads were assembled *de novo* following the Hierarchical Genome Assembly Process (HGAP3.0) approach (SMRT analysis version: 2.3.0, patch #4) for Pacific Bioscience using the *WGS-Celera Assembler* 7.0 (Myers et al., 2000) and Quiver algorithm (Chen-Shan Chin et al., 2013). Once assembled, the prediction of Coding DNA Sequences (CDS) was done with the help of the GenMark program (Besemer et al., 2001). Furthermore, prediction of tRNA, rRNA, and mRNA genes and signal peptides in the sequences was achieved using *tRNAscan* (version 2.0), *RNAmmer* (Version 1.2)*, HMMer* [HMMER 3.1 (July 2017)]^1^ programs, respectively (Lowe and Eddy, 1997; Lagesen et al., 2007). The assembled genome sequences were annotated using the *BLAST2go* program version 4.1.9 (Conesa et al., 2005) followed by a complementary annotation specific for protein domains using the *HMMer* program [HMMER 3.1 (July 2017)] see footnote ^2^ and Pfam database. Furthermore, the annotation process also included blasting genes against Clusters of Orthologous Groups (COGs) of proteins using the *WebMGA* server (Wu et al., 2010). The circular maps of chromosome and plasmids were performed by Artemis and DNAPlotter software (Carver et al., 2005, 2009).

Genome sequencing, assembly, and annotation were done at Biopolis (Valencia, Spain). The complete genome sequence of *L. pentosus* CF2-10 N was deposited at the EMBL Nucleotide Sequence Database (accession number of ERR11550479).

### Comparative genomic analysis of *Lactiplantibacillus pentosus* CF2-10 N and other *Lactiplantibacillus pentosus* strains

Genome sequences of *L. pentosus* CF2-10 N and other *L. pentosus* strains (MP-10, IG1 and KCA1) were aligned using MAUVE (Darling et al., 2004) available in DNASTAR Lasergene (version 17.3). Trees were then generated using RAxML with default parameters (Stamatakis, 2014). Further genome alignment and comparison of *L. pentosus* CF2-10 N and other *L. pentosus* strains (IG1 and KCA1) isolated from different ecological niches or *L. plantarum* WCFS1 (as reference strain) was done using the *MUMmer* program (version 3.0), considering alignment > 500 bp. The genome accession numbers of strains used in this study are as follows: *L. pentosus* IG1 (PRJEA67801), *L. pentosus* KCA1 (PRJNA81575, GenBank assembly accession GCA_000271445.1) and *L. plantarum* WCFS1 (PRJNA356, GenBank assembly accession GCA_000203855.3). Functional annotation of CDS (COG) for the three strains (*L. pentosus* IG1, *L. pentosus* KCA1 and *L. plantarum* WCFS1) was completed following the same strategy as for *L. pentosus* CF2-10 N by using reciprocal blast (*BLAST2go*) program version 4.1.9 (Conesa et al., 2005) and the available genome sequences in NCBI.

### Genomic analysis of safety aspects and defense mechanisms of *Lactiplantibacillus pentosus* CF2-10 N

For specific annotation of antibiotic resistance genes (ARGs), the Resistance Gene Identifier (RGI) software (as part of the CARD “The Comprehensive Antibiotic Resistance Database” tools; Alcock et al., 2020) was used for the prediction of the *L. pentosus* CF2-10 N resistome from protein or nucleotide data based on homology and SNP (Single Nucleotide Polymorphism) models, employing the CARD’s curated AMR (antimicrobial resistance) detection models (last accessed in March 2022). In addition, the genome of *L. pentosus* CF2-10 N was investigated for acquired antibiotic resistance genes/chromosomal mutations mediating antimicrobial resistance through the ResFinder^3^ software version 4.1 (Zankari et al., 2012; Bortolaia et al., 2020) with selected %ID threshold of 90.00% and selected minimum length of 60% (last accessed in March 2022).

Regarding virulence factors (VFs), the predicted CDSs were annotated using reciprocal BLAST against the Virulence Factors of Bacterial Pathogens (VFDB) database. Hits were considered positive when the results of reciprocal BLAST were similar, employing a 80% sequence similarity cut-off (Liu et al., 2019).

Concerning mobile genetic elements, the annotated genome sequence of *L. pentosus* CF2-10 N was screened for the presence of conjugative plasmid, transposase, transposon, IS elements and prophage coding genes. The genome was searched for Insertion Sequences (IS) using the ISfinder search tool (Zhang et al., 2000). Furthermore, complementary information on prophage DNA within the *L. pentosus* CF2-10 N genome was obtained by using bioinformatic tools such as PHASTER’s version (PHAge Search Tool Enhanced Release, last updated March 2016; corresponding to the updated prophage/virus database PHAST “PHAge Search Tool”) for the rapid identification and annotation of prophage sequences within bacterial genomes and plasmids (Zhou et al., 2011; Arndt et al., 2016).

Finally, the annotated genome sequence of *L. pentosus* CF2-10 N was screened for the presence of CRISPR (Clustered Regularly Interspaced Short Palindromic Repeats) coding genes and the localization of CRISPR RNAs targets was identified using the CRISPRDetect program version 2.4 (Biswas et al., 2016).^4^

### Genomic analysis of probiotic properties of *Lactiplantibacillus pentosus* CF2-10 N

To identify the putative genes associated with probiotic characteristics in *L. pentosus* CF2-10 N, the annotated genome sequence was screened for the presence of genes coding for proteins involved in cell adhesion (mucus binding proteins, cell surface proteins and moonlighting proteins among others), exopolysaccharide (EPS) biosynthesis, tolerance to low pH and bile salts, enzyme production, vitamin biosynthesis and host immunomodulation.

## Results

### General genomic features of a probiotic *Lactiplantibacilluis pentosus* CF2-10 N

**Figure 1:**
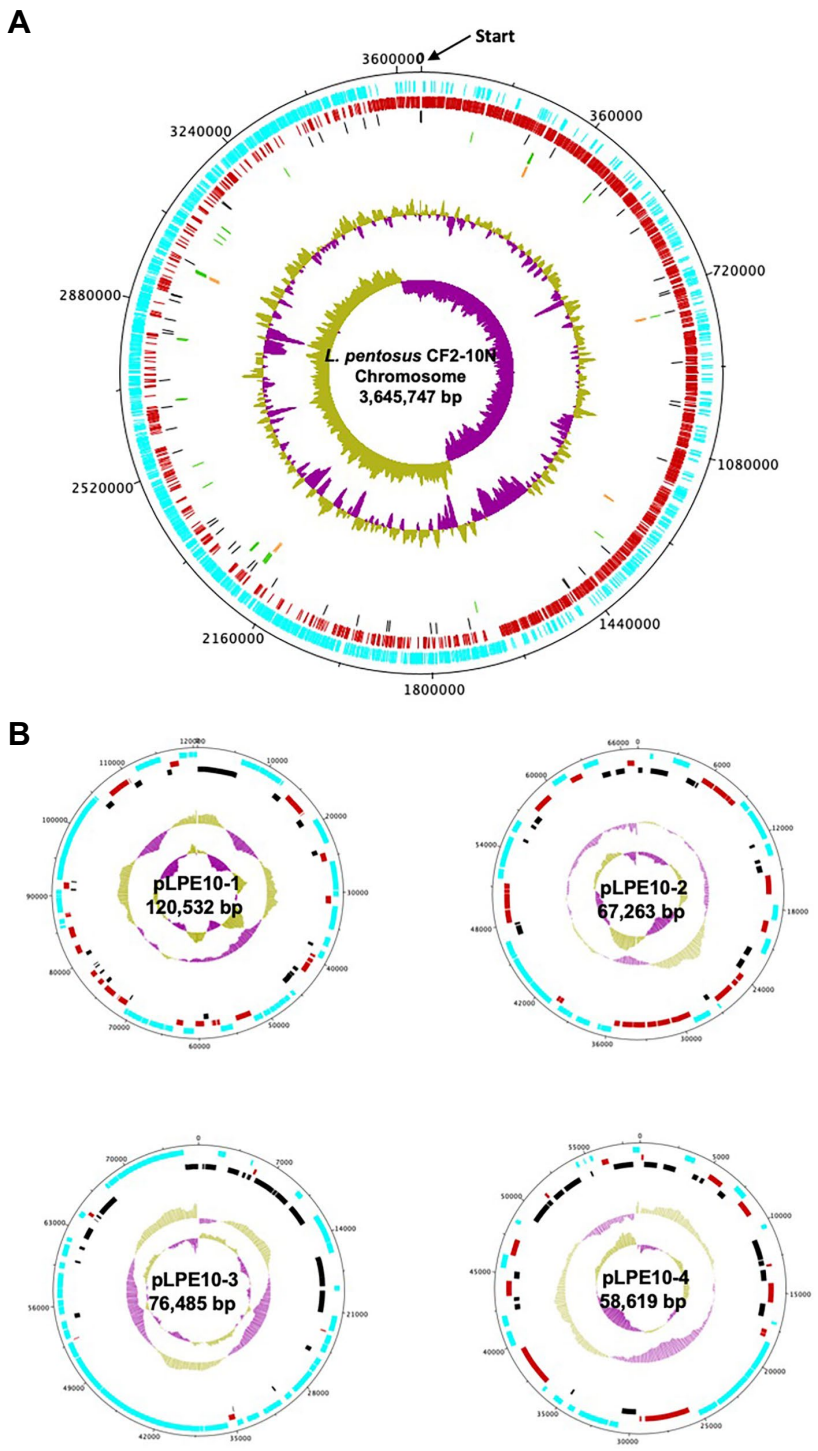
Circular map of the *Lactiplantibacillus pentosus* CF2-10N chromosome **(A)** and four plasmids **(B)**. The circles from outside to inside are the annotated Coding DNA Sequences (CDS) elements in forward orientation (blue); the annotated CDS elements in the reverse orientation (red); the Pseudogenes (black); the tRNA (green); the rRNA (orange); the %GC plot and the GC skew.

The analysis revealed that the *Lactiplantibacillus pentosus* CF2-10 N genome consisted of a single circular chromosome of 3,645,747 bp, with an estimated mol% G + C content of 46.42% and 4 plasmids ranging 58–120 kb (Figure 1). The annotated genome sequence (Figure 1) revealed 3,713 open reading frames (ORFs), of which 75.4% (2,801) were attributed to a COG (Cluster of Orthologous Groups) family and/or were given a functional description (Supplementary Table S1). Furthermore, 16 rRNA genes were predicted in *L. pentosus* CF2-10 N genome using *RNAmmer* (version 1.2), while 67 tRNA encoding sequences were identified corresponding to all 20 amino acids and three undermined amino acids (Supplementary Table S2).

Supplementary Figure S1 shows the biological processes, the cellular components and the molecular function frequencies predicted in L. pentosus CF2-10 N. Among the Gene Ontology (GO) terms, those related to biological processes such as oxidation–reduction process, regulation of transcription, DNA-templated transcription and DNA-templated transmembrane transport were the most identified. Regarding molecular function, ATP-binding and DNA binding were the most prevalent. However, in both biological process and molecular function about 1,250–1,550 genes have no known biological process/function (Supplementary Figure S1).

The most abundant COG category of *L. pentosus* CF2-10 N genome, except for “[S] Function unknown” (273 CDSs, 9.7%), was “[R] General function prediction only” (336 CDSs, 12%), followed by “[G] Carbohydrate transport and metabolism” (307 CDSs, 11%), “[K] Transcription” (235 CDSs, 8.4%), “[L] Replication, recombination and repair” (213 CDSs, 7.6%) and “[E] Amino acid transport and metabolism” (192 CDSs, 6.9%), accounting for 45.9% of the overall CDS (1,283/2,801 CDSs; Supplementary Table S3).

### Comparative genome analysis of *Lactiplantibacillus pentosus* CF2-10 N

**Figure 2:**
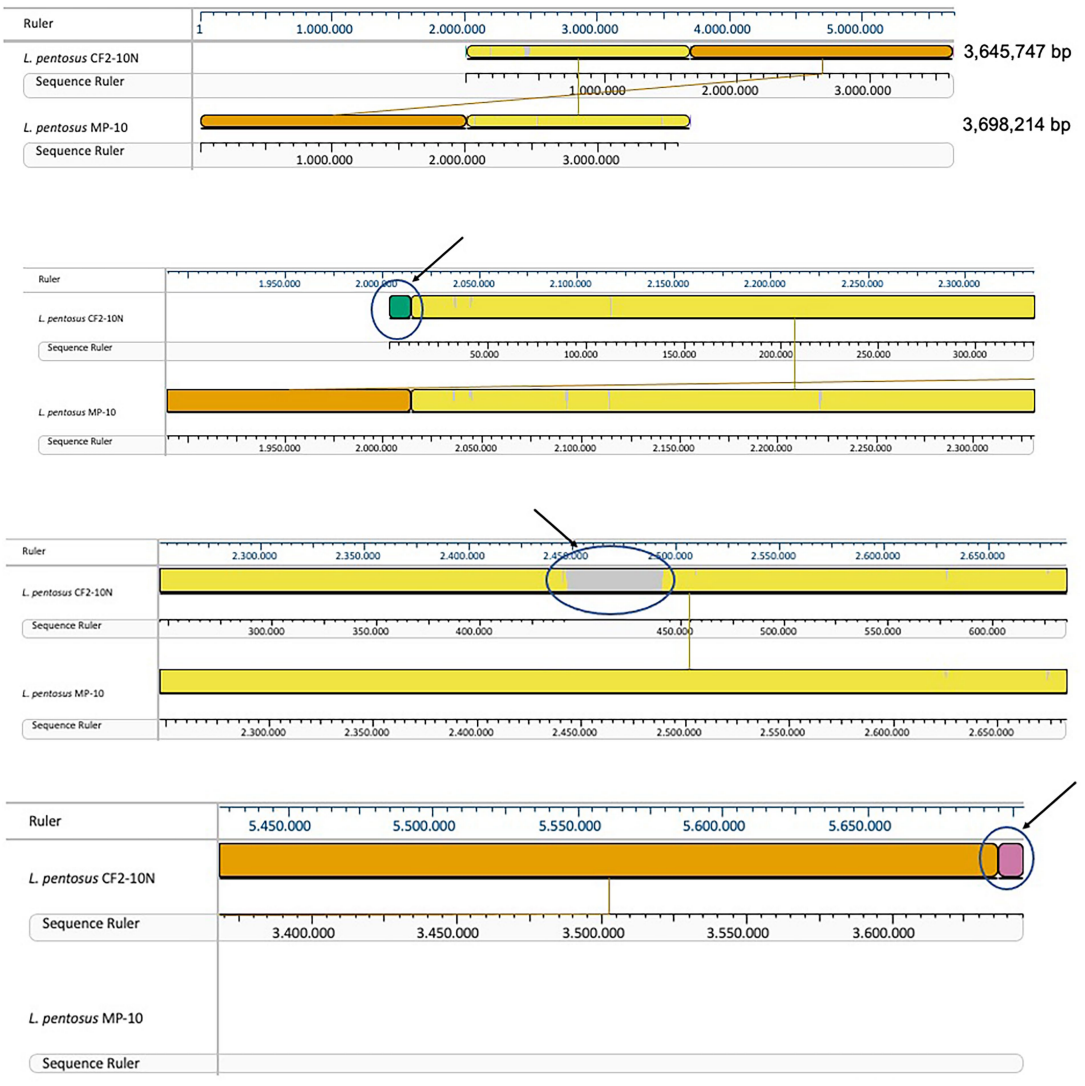
Mauve visualization of whole genome alignment of *L. pentosus* CF2-10N with *L. pentosus* MP-10.

Comparative genomic analysis of *L. pentosus* CF2-10 N and *L. pentosus* MP-10 isolated from the same ecological niche (Aloreña table olives) showed that both *L. pentosus* strains shared 99.87% identity as revealed by sequence alignment using the MAUVE algorithm. This high similarity was further highlighted by large blocks of colinearization in the MAUVE alignment, being the synteny of genes similar, although inversion, insertion and rearrangement occurred (Figure 2). Besides *L. pentosus* MP-10 (isolated from Aloreña table olives), comparison with other *L. pentosus* strains by genome sequence alignment (using MAUVE), notably IG1 (isolated from olives) and KCA1 (isolated from the vaginal tract), revealed genetic differences among the studied strains (Supplementary Figure S2A). To illustrate this relationship, a maximum-likelihood core genome tree was constructed using RaxML which showed higher phylogenetic similarity in the case of *L. pentosus* CF2-10 N and MP-10 strains (evolutionary distance “ED,” ED = 0), followed by *L. pentosus* IG1 (ED = 0.02) and then *L. pentosus* KCA1 (ED = 0.08; Supplementary Figure S2B).

**Figure 3:**
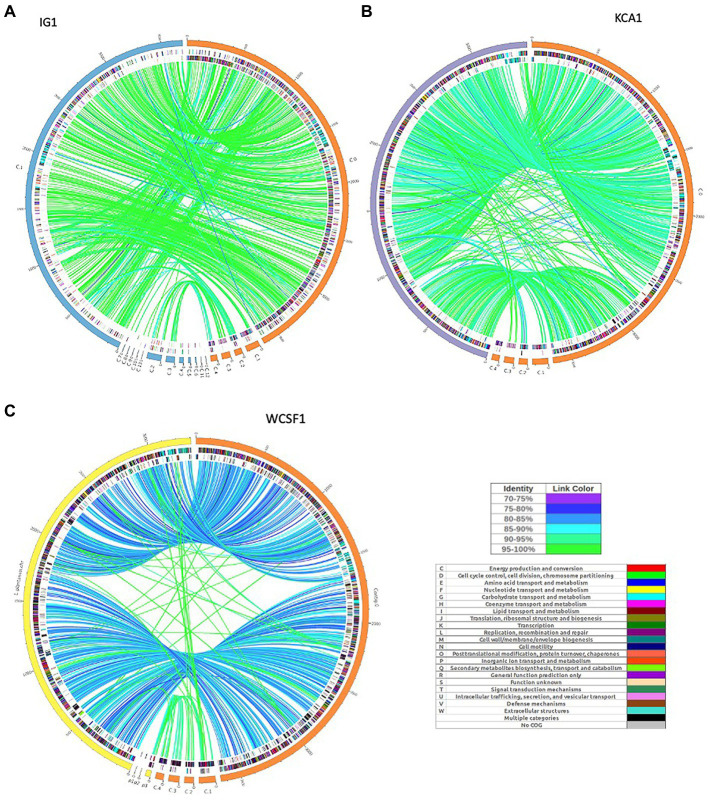
Circos map showing genome synteny between the genetic linkage map of *L. pentosus* CF2-10N and the reference genome sequences: *L. pentosus* IG1 **(A)**, *L. pentosus* KCA1 **(B)** or *L. plantarum* WCFS1 **(C)**. Color synteny linkages were generated using Circos. Rings from outside to inside are genomes of *L. pentosus* CF2-10 N (orange) and *L. pentosus* IG1 (blue), *L. pentosus* KCA1 (purple) or *L. plantarum* WCFS1 (yellow); shared Cluster of Orthologous Groups of proteins (COG) annotated coding sequences between *L. pentosus* CF2-10 N and the reference strain as analyzed by reciprocal blast (*BLAST2go*) colored by their COG annotation; and the unique Cluster of Orthologous Groups of proteins (COG) annotated coding sequences of each genome (*L. pentosus* CF2-10 N and the reference strain).

**Figure 4:**
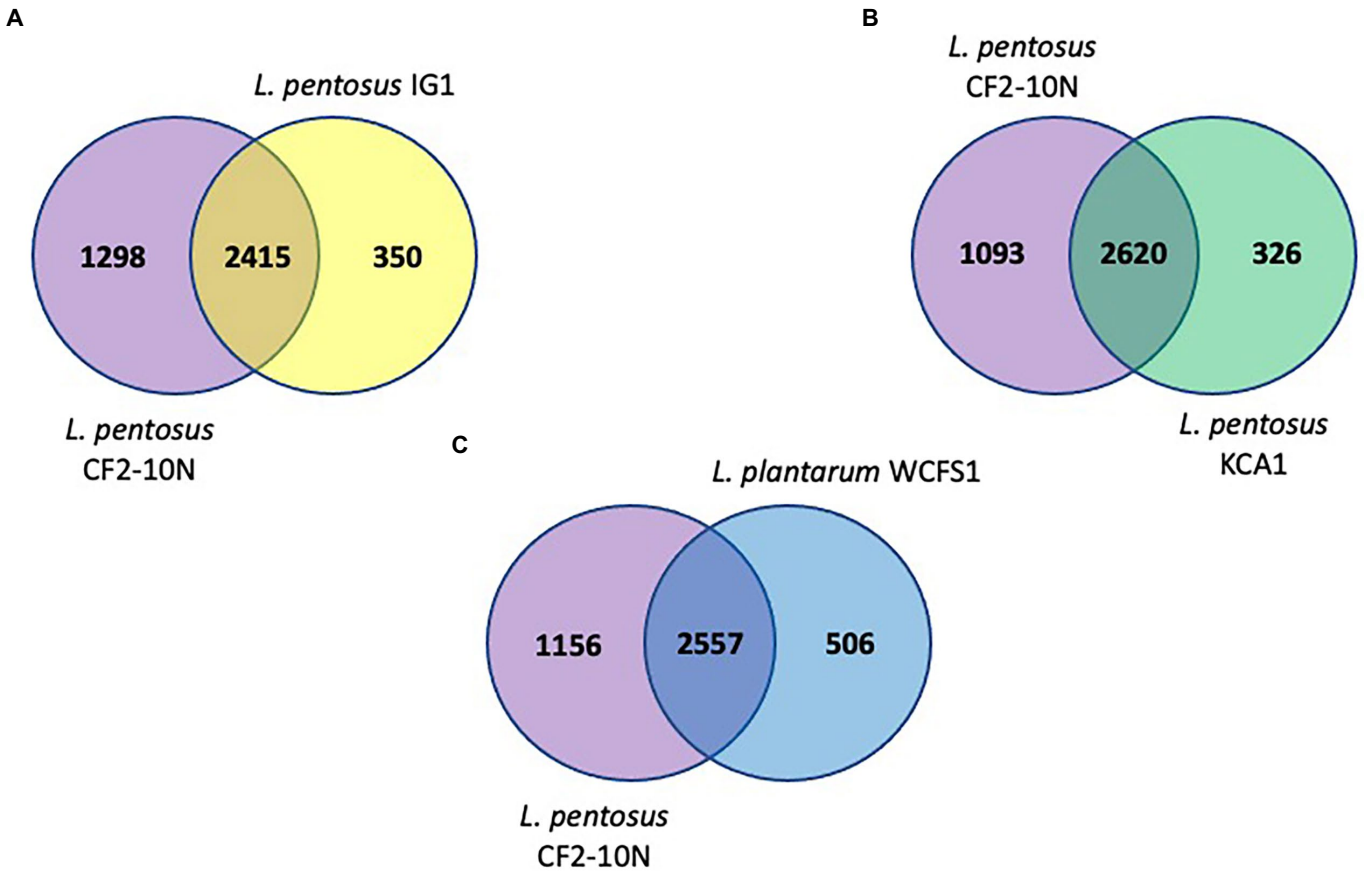
Venn diagrams showing number of reciprocal best hits among *L. pentosus* CF2-10 N and other lactobacilli subset of core genomes. **(A)** Number of shared and unique genes of core genomes of *L. pentosus* CF2-10N and *L. pentosus* IG1. **(B)** Number of shared and unique genes of core genomes of *L. pentosus* CF2-10N and *L. pentosus* KCA1. **(C)** Number of shared and unique genes of core genomes of *L. pentosus* CF2-10N and *L. plantarum* WCFS1.

The synteny linkage of *L. pentosus* CF2-10 N against *L. pentosus* IG1 and KCA1 strains or *L. plantarum* WCFS1 was further analyzed using the *MUMmer* program and represented using Circos (Figure 3; Supplementary Tables S4–S6). Here, the genome comparison revealed the presence of highly conserved syntenic blocks between *L. pentosus* strains (IG1 and KCA1; Figures 3A,B), and to a lesser extent with *L. plantarum* WCFS1 (Figure 3C). On the other hand, comparison of the number of unique and shared annotated genes of *L. pentosus* CF2-10 N and other strains (*L. pentosus* IG1, *L. pentosus* KCA1 or *L. plantarum* WCFS1) using reciprocal blast revealed unique genomic features in *L. pentosus* CF2-10 N (Figure 4).

Finally, *L. pentosus* CF2-10 N appears to share both core and accessory annotated genes with *L. pentosus* KCA1 (88.93% hits, Figure 4B) and *L. pentosus* IG1 (87.34% hits; Figure 4A) and to a slightly lesser extent with *L. plantarum* WCFS1 (83.48%, Figure 4C).

### *In silico* analysis of safety determinants and defense mechanisms of *Lactiplantibacillus pentosus* CF2-10 N

Safety properties are a crucial feature of potential probiotic strains and their determination is considered a priority when characterizing a new potential probiotic. Hence, in a first step, antibiotic resistance and virulence determinants were screened in the *L. pentosus* CF2-10 N genome sequence. To do so, *in silico* prediction of antibiotic resistance genes (ARG) was done against the Comprehensive Antibiotic Resistance Database (CARD) using the RGI tool v3.2.1 available in the CARD database^5^ which used archive’s curated AMR (antimicrobial resistance) detection models. Results indicated no ARG in the *L. pentosus* CF2-10 N genome sequence. Thus, neither resistance genes nor mutations conferring antibiotic resistance was predicted in the complete resistome of *L. pentosus* CF2-10 N. However *BLAST2go* annotation revealed the presence of non-specific antimicrobial resistance mechanisms relying on efflux transporters or transmembrane proteins involved in response to antibiotics such as ABC transporter ATP-binding protein (encoded by *LPE_03051*, *LPE_00789*, *FD24_GL000501* genes), TIGR00374 family protein (encoded by *mprF* gene), undecaprenyl-diphosphatase (encoded by *uppP* gene), QacE family quaternary ammonium compound efflux SMR transporter (encoded by *FD24_GL003284* gene), MATE family efflux transporter (encoded by *LPE_00986* gene) and cation efflux pump (encoded by *FD24_GL002035* gene).

With regard to acquired resistance by horizontal gene transfer, ResFinder did not detect any acquired antibiotic resistance genes for aminoglycoside, beta-lactam, colistin, disinfectant, fluoroquinolone, fosfomycin, fusidic acid, glycopeptide, MLS-series (Macrolide, lincosamide and streptogramin B), nitroimidazole, oxazolidinone, phenicol, pseudomonic acid, rifampicin, sulphonamide, tetracycline and trimethoprim (data not shown).

Regarding virulence, the predicted CDSs annotated using reciprocal BLAST against VFDB (database including only experimentally validated virulence factors) did not identify any known virulence factors including toxins.

Analysis of the *L. pentosus* CF2-10 N mobilome showed that the bacterial genome included 66 transposases: 19 transposases, 1 transposase family protein A and 46 transposases belonging to nine IS transposase families (4 IS3, 6 IS5, 5 IS21, 17 IS30, 4 IS66, 3 IS1380, 2 ISL3, 2 DDE, 2 IS6501, 1 IS200/IS605), mainly located on plasmids (pLPE10-1, pLPE10-2 and pLPE10-4) rather than on the chromosome (50 on plasmids/16 on chromosome) and appearing in multiple copies ranging from two to five (Table 1). IS30 family transposases were abundant (17 of 66 transposases) and were represented by seven different genes (Table 1). Furthermore, Blastp alignment of transposase protein sequences detected in *L. pentosus* CF2-10 N genome showed high similarity with *L. pentosus* (29 of 66 transposases, 98.9–100%), *L. plantarum* (11 of 66 transposases, 95.2–100%) and other lactobacilli. It is noteworthy to indicate the presence of 34 paired (adjacent to each other in the genome) transposase genes (2 or 3 genes) being different genes or belonging to different families and located on both chromosome and plasmids (Table 1). Regarding IS elements, 45 CDS were predicted distributed into 16 different families and in various bacteria (Table 2). Here, IS30 and IS3 were the most detected elements followed by IS5 (Table 2).

**Table 1:** Characterization of transposases predicted in the *Lactiplantibacillus pentosus* CF2-10N genome.

Gene ID	Gene	Position	Strand	Gene length	Protein description	COG ID (COG description)	COG class (COG class description)	Similarity to transposase in Lactiplantibacillus^*^
gene_86	*gene_86*	89,400-90,662	+	1,263	ISL3 family transposase	–	−	99.3% *L. pentosus*
gene_204	*LPENT_00003*	219,476–220,444	−	969	MULTISPECIES: IS30 family transposase	COG2826 (Transposase and inactivated derivatives, IS30 family)	L (Replication, recombination and repair)	100% Lactobacillaceae
gene_638	*gene_638*	700,336–701,475	−	1,140	Transposase	COG0675 (Transposase and inactivated derivatives)	L (Replication, recombination and repair)	100% *L. pentosus*
gene_639	*LPE_01510*	701,456–701,908	−	453	Transposase family protein A	COG1943 (Transposase and inactivated derivatives)	L (Replication, recombination and repair)	100% *L. pentosus*
gene_700	*FD14_GL001685*	761,249–762,559	−	1,311	IS1380 family transposase	−	−	100% *L. pentosus*
gene_1236	*gene_1236*	1,345,052–1,345,423	+	372	IS5 family transposase	COG3293 (Transposase and inactivated derivatives)	L (Replication, recombination and repair)	100% *P. acidilactici*
gene_1237	*gene_1237*	1,345,396–1,345,827	+	432	Putative transposase for insertion sequence element IS6501	COG3293 (Transposase and inactivated derivatives)	L (Replication, recombination and repair)	97.9% *L. plantarum*
gene_2023	*tnp1*	2,236,745–2,237,668	−	924	MULTISPECIES: IS30 family transposase	COG2826 (Transposase and inactivated derivatives, IS30 family)	L (Replication, recombination and repair)	100% Terrabacteria group
gene_2025	*FD24_GL002607*	2,239,842–2,240,885	+	1,044	MULTISPECIES: IS30 family transposase	COG2826 (Transposase and inactivated derivatives, IS30 family)	L (Replication, recombination and repair)	99.7% *Lactiplantibacillus*
gene_2321	*HR47_01150*	2,551,136–2,552,023	+	888	IS30 family transposase	COG2826 (Transposase and inactivated derivatives, IS30 family)	L (Replication, recombination and repair)	100% Lactobacillaceae
gene_2680	*FD14_GL001685*	2,918,782–2,920,092	−	1,311	IS1380 family transposase	−	−	100% *L. pentosus*
gene_2,707	*HR47_01150*	2,948,652–2,949,539	+	888	IS30 family transposase	COG2826 (Transposase and inactivated derivatives, IS30 family)	L (Replication, recombination and repair)	100% Lactobacillaceae
gene_2843	*HR47_01150*	3,146,814–3,147,701	+	888	IS30 family transposase	COG2826 (Transposase and inactivated derivatives, IS30 family)	L (Replication, recombination and repair)	100% Lactobacillaceae
gene_3192	*gene_3192*	3,516,037–3,516,375	+	339	MULTISPECIES: IS5 family transposase	COG3293 (Transposase and inactivated derivatives)	L (Replication, recombination and repair)	100% Bacilli
gene_3261	gene_3261	3,595,253–3,596,521	−	1,269	Transposase	COG0675 (Transposase and inactivated derivatives)	L (Replication, recombination and repair)	100% *L. pentosus*
gene_3262	*LPE_00194*	3,596,619–3,597,059	+	441	IS200/IS605 family transposase	COG1943 (Transposase and inactivated derivatives)	L (Replication, recombination and repair)	100% *L. pentosus*
gene_3455^£^	*FD47_GL000486*	24,260-25,474	−	1,215	IS21 family transposase	COG4584 (Transposase and inactivated derivatives)	L (Replication, recombination and repair)	100% *Lactiplantibacillus*
gene_3464^£^	*FD14_GL001685*	36,418-37,728	+	1,311	IS1380 family transposase	−	−	100% *L. pentosus*
gene_3465^£^	*LPE_03103*	38,516-39,514	+	999	IS30 family transposase	COG2826 (Transposase and inactivated derivatives, IS30 family)	L (Replication, recombination and repair)	100% *L. pentosus*
gene_3484^£^	*LSEI_2008*	53,623-54,552	−	930	MULTISPECIES: IS30 family transposase	COG2826 (Transposase and inactivated derivatives, IS30 family)	L (Replication, recombination and repair)	99.7% Lactobacillales
gene_3486^£^	*gene_3486*	55,300-57,009	+	1710	DDE transposase	COG3666 (Transposase and inactivated derivatives)	L (Replication, recombination and repair)	98.9% *L. plantarum*
gene_3490^£^	*FD47_GL000486*	59,298-60,512	−	1,215	IS21 family transposase	COG4584 (Transposase and inactivated derivatives)	L (Replication, recombination and repair)	100% *Lactiplantibacillus*
gene_3492^£^	*LPENT_00003*	62,337-63,305	−	969	MULTISPECIES: IS30 family transposase	COG2826 (Transposase and inactivated derivatives, IS30 family)	L (Replication, recombination and repair)	100% Lactobacillaceae
gene_3505^£^	*gene_3505*	73,427-75,076	−	1,650	DDE transposase	COG3666 (Transposase and inactivated derivatives)	L (Replication, recombination and repair)	99.5% *L. plantarum*
gene_3506^£^	*gene_3506*	75,204-75,746	−	543	Transposase	COG3666 (Transposase and inactivated derivatives)	L (Replication, recombination and repair)	100% *L. pentosus*
gene_3507^£^	*gene_3507*	75,794-76,501	−	708	Transposase, partial	COG2963 (Transposase and inactivated derivatives)	L (Replication, recombination and repair)	100% *L. pentosus*
gene_3509^£^	*gene_3509*	77,298-77,729	−	432	Putative transposase for insertion sequence element IS6501	COG3293 (Transposase and inactivated derivatives)	L (Replication, recombination and repair)	97.9% *L. plantarum*
gene_3510^£^	*gene_3510*	77,702-78,073	−	372	IS5 family transposase	COG3293 (Transposase and inactivated derivatives)	L (Replication, recombination and repair)	100% *P. acidilactici*
gene_3519^£^	*FD47_GL002738*	83,726-84,565	−	840	ISSth1, transposase (Orf2), IS3 family	COG2801 (Transposase and inactivated derivatives)	L (Replication, recombination and repair)	100% *L. pentosus*
gene_3520^£^	*LPENT_00063*	84,601-85,323	−	723	Transposase (transposase, IS3 family protein)	COG2963 (Transposase and inactivated derivatives)	L (Replication, recombination and repair)	100% *L. pentosus*
gene_3530^£^	*LPENT_00003*	91,009-91,977	−	969	MULTISPECIES: IS30 family transposase	COG2826 (Transposase and inactivated derivatives, IS30 family)	L (Replication, recombination and repair)	100% Lactobacillaceae
gene_3557^£^	*FD47_GL000486*	116,556-117,770	−	1,215	IS21 family transposase	COG4584 (Transposase and inactivated derivatives)	L (Replication, recombination and repair)	100% *Lactiplantibacillus*
gene_3565^§^	*gene_3565*	4,191-4,487	+	297	Transposase (plasmid)	−	−	100% *L. plantarum*
gene_3566^§^	*gene_3566*	4,782-5,018	+	237	Transposase (plasmid)	COG3464 (Transposase and inactivated derivatives)	L (Replication, recombination and repair)	100% *L. plantarum*
gene_3568^§^	*FC27_GL001295*	6,430-6,975	+	546	Transposase	COG3328 (Transposase and inactivated derivatives)	L (Replication, recombination and repair)	98.9% *L. paraplantarum*
gene_3569^§^	*LPENT_00125*	7,062-7,991	+	930	Transposase TraISLpl1 (IS30 family)	COG2826 (Transposase and inactivated derivatives, IS30 family)	L (Replication, recombination and repair)	99.7% *L. pentosus*
gene_3574^§^	*gene_3574*	10,980-11,261	−	282	Transposase IS66	−	−	98.9% *L. pentosus*
gene_3576^§^	*gene_3576*	11,507-12,520	−	1,014	IS66 family transposase	COG3436 (Transposase and inactivated derivatives)	L (Replication, recombination and repair)	100% *L. pentosus*
gene_3577^§^	*gene_3577*	12,731-12,940	−	210	MULTISPECIES: transposase	COG3436 (Transposase and inactivated derivatives)	L (Replication, recombination and repair)	100% *L. pentosus*
gene_3579^§^	*gene_3579*	13,597-14,094	+	498	Transposase	COG3293 (Transposase and inactivated derivatives)	L (Replication, recombination and repair)	100% *L. pentosus*
gene_3603^§^	*gene_3603*	36,269-36,484	+	216	MULTISPECIES: transposase	COG3464 (Transposase and inactivated derivatives)	L (Replication, recombination and repair)	100% *L. pentosus*
gene_3607^§^	*FC99_GL000344*	40,024-40,968	+	945	IS30 family transposase	COG2826 (Transposase and inactivated derivatives, IS30 family)	L (Replication, recombination and repair)	100% *L. pentosus*
gene_3609^§^	*gene_3609*	42,455-42,751	+	297	Transposase (plasmid)	−	−	100% *L. plantarum*
gene_3610^§^	*gene_3610*	43,046-43,282	+	237	Transposase (plasmid)	COG3464 (Transposase and inactivated derivatives)	L (Replication, recombination and repair)	100% *L. plantarum*
gene_3612^§^	*FC27_GL001295*	44,694-45,239	+	546	Transposase	COG3328 (Transposase and inactivated derivatives)	L (Replication, recombination and repair)	98.9% *L. paraplantarum*
gene_3613^§^	*LPENT_00125*	45,326-46,255	+	930	Transposase TraISLpl1 (IS30 family)	COG2826 (Transposase and inactivated derivatives, IS30 family)	L (Replication, recombination and repair)	99.7% *L. pentosus*
gene_3619^§^	*gene_3619*	49,243-49,524	−	282	Transposase IS66	−	−	98.9% *L. pentosus*
gene_3621^§^	*gene_3621*	50,051-50,782	−	732	IS66 family transposase	COG3436 (Transposase and inactivated derivatives)	L (Replication, recombination and repair)	100% *L. pentosus*
gene_3622^§^	*gene_3622*	50,992-51,201	−	210	MULTISPECIES: transposase	COG3436 (Transposase and inactivated derivatives)	L (Replication, recombination and repair)	100% *L. pentosus*
gene_3624^§^	*gene_3624*	51,857-52,252	+	396	Transposase	COG3293 (Transposase and inactivated derivatives)	L (Replication, recombination and repair)	100% *L. pentosus*
gene_3642^&^	*gene_3642*	4,323-4,721	−	399	Transposase	COG3293 (Transposase and inactivated derivatives)	L (Replication, recombination and repair)	100% *L. plantarum*
gene_3643^&^	*gene_3643*	4,784-5,122	−	339	MULTISPECIES: IS5 family transposase	COG3293 (Transposase and inactivated derivatives)	L (Replication, recombination and repair)	100% Bacilli
gene_3652^&^	*gene_3652*	12,866-13,795	+	930	IS30 family transposase	COG2826 (Transposase and inactivated derivatives, IS30 family)	L (Replication, recombination and repair)	99.3% *L. pentosus*
gene_3658^&^	*HR47_01150*	18,998-19,885	−	888	IS30 family transposase	COG2826 (Transposase and inactivated derivatives, IS30 family)	L (Replication, recombination and repair)	100% Lactobacillaceae
gene_3659^&^	*FD47_GL000486*	20,362-21,576	+	1,215	IS21 family transposase	COG4584 (Transposase and inactivated derivatives)	L (Replication, recombination and repair)	100% *Lactiplantibacillus*
gene_3663^&^	*gene_3663*	23,236-23,574	−	339	MULTISPECIES: IS5 family transposase	COG3293 (Transposase and inactivated derivatives)	L (Replication, recombination and repair)	100% Bacilli
gene_3667^&^	*gene_3667*	26,120-26,794	−	675	Transposase	COG3415 (Transposase and inactivated derivatives)	L (Replication, recombination and repair)	100% *Loigolactobacillus*
gene_3669^&^	*FD00_GL002377*	27,785-29,125	+	1,341	ISL3 family transposase ISLasa4c	COG3464 (Transposase and inactivated derivatives)	L (Replication, recombination and repair)	100% *Liquorilactobacillus uvarum*
gene_3670^&^	*FD47_GL002738*	29,250-30,089	−	840	ISSth1, transposase (Orf2), IS3 family	COG2801 (Transposase and inactivated derivatives)	L (Replication, recombination and repair)	100% *L. pentosus*
gene_3671^&^	*LPENT_00063*	30,125-30,847	−	723	Transposase (transposase, IS3 family protein)	COG2963 (Transposase and inactivated derivatives)	L (Replication, recombination and repair)	100% *L. pentosus*
gene_3689^&^	*gene_3689*	46,896-47,294	−	399	Transposase	COG3293 (Transposase and inactivated derivatives)	L (Replication, recombination and repair)	100% *L. plantarum*
gene_3690^&^	*gene_3690*	47,357-47,695	−	339	MULTISPECIES: IS5 family transposase	COG3293 (Transposase and inactivated derivatives)	L (Replication, recombination and repair)	100% Bacilli
gene_3699^&^	*gene_3699*	55,438-56,367	+	930	IS30 family transposase	COG2826 (Transposase and inactivated derivatives, IS30 family)	L (Replication, recombination and repair)	99.4% *L. pentosus*
gene_3705^&^	*HR47_01150*	61,570-62,457	−	888	IS30 family transposase	COG2826 (Transposase and inactivated derivatives, IS30 family)	L (Replication, recombination and repair)	100% Lactobacillaceae
gene_3706^&^	*FD47_GL000486*	62,934-64,148	+	1,215	IS21 family transposase	COG4584 (Transposase and inactivated derivatives)	L (Replication, recombination and repair)	100% *Lactiplantibacillus*
gene_3710^&^	*gene_3710*	65,413-65,592	−	180	Transposase	−	−	95.2% *L. plantarum*

* : the best hit was indicated.

£ : sequences of pLPE10-1 plasmid.

§ : sequences of pLPE10-4 plasmid.

& : sequences of pLPE10-2 plasmid.

**Table 2:** Characterization of IS elements found within the genome of *Lactiplantibacillus pentosus* CF2-10 N using the ISfinder search tool.

Sequences producing significant alignments	IS Family	Group	Origin	Score (bits)	*E* value
ISP1	ISL3		*Lactobacillus plantarum*	2,547	0.0
ISLdl3	IS30		*Lactobacillus delbrueckii*	1705	0.0
ISLhe30	IS30		*Lactobacillus helveticus*	1,635	0.0
ISLpl3	IS5	IS427	*Lactobacillus plantarum*	1,429	0.0
ISLsa1	IS30		*Lactobacillus sakei*	494	6e-136
ISLpl2	IS3	IS150	*Lactobacillus plantarum*	56.0	4e-04
ISLhe65	IS200/IS605	IS1341	*Lactobacillus helveticus*	54.0	0.002
ISP2	IS1182		*Lactobacillus plantarum*	52.0	0.007
ISMmu1	IS200/IS605	IS605	*Mitsuokella multacida*	52.0	0.007
ISLjo5	IS200/IS605	IS605	*Lactobacillus johnsonii*	52.0	0.007
ISSpn5	IS1380		*Streptococcus pneumoniae*	50.1	0.026
IS1161	IS30		*Streptococcus salivarius*	48.1	0.10
IS1139	IS30		*Streptococcus salivarius*	48.1	0.10
IS1086	IS30		*Ralstonia eutropha*	48.1	0.10
ISRhru6	IS5	IS1031	*Rhodospirillum rubrum*	46.1	0.41
ISAar45	IS3	IS3	*Arthrobacter arilaitensis*	46.1	0.41
ISMsm7	IS3	IS3	*Mycobacterium smegmatis*	46.1	0.41
IS6770	IS30		*Enterococcus faecalis*	46.1	0.41
IS1648	IS5	IS427	*Streptomyces coelicolor*	46.1	0.41
ISAcba1	IS1595	ISSod11	*Actinobacteria bacterium*	44.1	1.6
ISBsp5	IS1182		*Bacillus* sp.	44.1	1.6
ISBam1	IS3	IS150	*Burkholderia ambifaria*	44.1	1.6
ISLrh4	ISLre2		*Lactobacillus rhamnosus*	44.1	1.6
ISSav4	IS701		*Streptomyces avermitilis*	44.1	1.6
IS231J	IS4	IS231	*Bacillus thuringiensis*	44.1	1.6
ISCfr26	IS110	IS1111	*Citrobacter freundii*	42.1	6.4
ISAbe16	IS3	IS150	*Acinetobacter bereziniae*	42.1	6.4
ISVat2	IS256	IS1249	*Veillonella atypica*	42.1	6.4
ISDha15	IS1634		*Desulfitobacterium dichloroeliminans*	42.1	6.4
ISPan1	IS5	IS903	*Pantoea ananatis*	42.1	6.4
ISPph2	IS630		*Pelodictyon phaeoclathratiforme*	42.1	6.4
ISShes12	IS1634		*Shewanella sp.*	42.1	6.4
ISSoc13	IS5	IS427	*Synechococcus sp.*	42.1	6.4
ISEnfa364	IS30		*Enterococcus faecalis*	42.1	6.4
ISNwi3	IS1595	ISNwi1	*Nitrobacter winogradskyi*	42.1	6.4
ISMma18	IS1634		*Methanosarcina mazei*	42.1	6.4
ISCfe1	IS607		*Campylobacter fetus*	42.1	6.4
ISLpl1	IS30		*Lactobacillus plantarum*	42.1	6.4
IS987	IS3	IS51	*Mycobacterium bovis*	42.1	6.4
IS986	IS3	IS51	*Mycobacterium tuberculosis*	42.1	6.4
IS6110	IS3	IS51	*Mycobacterium tuberculosis*	42.1	6.4
IS231B	IS4	IS231	*Bacillus thuringiensis*	42.1	6.4
IS231A	IS4	IS231	*Bacillus thuringiensis*	42.1	6.4
IS231K	IS4	IS231	*Bacillus cereus*	42.1	6.4
IS1476	ISL3		*Enterococcus faecium*	42.1	6.4

On the other hand, screening for prophage DNA within the *L. pentosus* CF2-10 N genome, using bioinformatic tools such as PHASTER, determined the presence of eight temperate phage regions (Table 3). Two regions were intact (Regions 2 and 3, score > 90), the other three were questionable (Regions 5, 6 and 7, score 70 ± 90), and the last three regions were incomplete (Regions 1, 4 and 8, score < 70). The complete prophage regions of the *L. pentosus* CF2-10 N chromosome were identified as *Lactobacillus* phage Sha1 (Regions 2 and 3; GC content, 41.55–41.88%; region length, 39.9–47.7 kb). Regarding the questionable prophage regions, they corresponded to *Staphylococcus* phage SP beta-like (Regions 5 and 6; GC content, 34.83–40.70%; region length, 13.7–19.4 kb) and *Escherichia* phage 500,465–1 (Region 7; GC content, 41.54%; region length, 18.8 kb). With respect to the incomplete prophage region, we identified three regions corresponding to *Lactobacillus* phage PLE3 (Region 1; GC content, 41.26%; region length, 15 kb), Enterobacteria phage fiAA91-ss (Region 4; GC content, 38.27%; region length, 23.4 kb) and *Escherichia* phage 500,465–1 (Region 8; GC content, 31.68%; region length, 6.7 kb; Table 3).

**Table 3:** Description of prophage regions detected in the *Lactiplantibacillus pentosus* CF2-10N genome by using the PHASTER bioinformatic tool.

Region	Region length	Completeness*	Score	Region position	Localization	Most common phage	GC%	Total proteins
1	15 kb	Incomplete	30	2,227,200–2,242,287	Chromosome	PHAGE_Lactob_PLE3_NC_031125(1)	41.26	11
2	39.9 kb	Intact	150	2,260,786–2,300,777	Chromosome	PHAGE_Lactob_Sha1_NC_019489(26)	41.55	54
3	47.7 kb	Intact	150	2,808,177–2,855,881	Chromosome	PHAGE_Lactob_Sha1_NC_019489(22)	41.88	68
4	23.4 kb	Incomplete	60	39,960–63,408	pLPE10-1	PHAGE_Entero_fiAA91_ss_NC_022750(2)	38.27	22
5	13.7 kb	Questionable	80	309–14,094	pLPE10-4	PHAGE_Staphy_SPbeta_like_NC_029119(2)	34.83	26
6	19.4 kb	Questionable	90	34,863–54,348	pLPE10-4	PHAGE_Staphy_SPbeta_like_NC_029119(2)	40.70	38
7	18.8 kb	Questionable	80	17,673–36,515	pLPE10-2	PHAGE_Escher_500,465_1_NC_049342(3)	41.54	22
8	6.7 kb	Incomplete	40	60,245–66,978	pLPE10-2	PHAGE_Escher_500,465_1_NC_049342(3)	31.68	10

* : Intact (score > 90), Questionable (score 70 ± 90), Incomplete (score < 70).

pLPE10: plasmid of *L. pentosus* CF2-10.

Among the defense mechanisms revealed by *in silico* analysis of the *L. pentosus* CF2-10 N genome sequence, CRISPR I and II systems (both signature genes for the Type I “*cas3*” and Type II “*cas9*” systems) were detected as defense response to mobile genetic elements (i.e., viruses, transposable elements and conjugative plasmids; Table 4). In this sense, 13 genes were identified as CRISPR associated protein responsible genes (*cas* genes) organized in two operons (Supplementary Figure S3), and six of them were new genes found in the *L. pentosus* CF2-10 N genome (Table 4). Regarding CRISPR arrays (CR), five CRISPR unquestionable arrays were identified by using the CRISPRDetect program and they are distributed throughout the genome sequence between 1,791,840 and 3,235,959 bp (Table 5).

**Table 4:** Characterization CRISPR associated proteins predicted in the *Lactiplantibacillus pentosus* CF2-10N genome.

Gene ID	Gene	Position	Strand	Gene length (bp)	Protein description	Ontology ID	Ontology term
gene_1618	*cas9*	1,785,693–1,789,037	+	3,345	Type II CRISPR RNA-guided endonuclease Cas9	GO:0003677, GO:0003723, GO:0004519, GO:0046872, GO:0043571, GO:0051607, GO:0090305	DNA binding, RNA binding, endonuclease activity, metal ion binding, maintenance of CRISPR repeat elements, defense response to virus, nucleic acid phosphodiester bond hydrolysis
gene_1619	*cas9*	1,789,043–1,789,759	+	717	Type II CRISPR RNA-guided endonuclease Cas9	GO:0003677, GO:0003723, GO:0004519, GO:0046872, GO:0043571, GO:0051607, GO:0090305	DNA binding, RNA binding, endonuclease activity, metal ion binding, maintenance of CRISPR repeat elements, defense response to virus, nucleic acid phosphodiester bond hydrolysis
gene_1620	*cas1*	1,789,953–1,790,858	+	906	Subtype II CRISPR-associated endonuclease Cas1	GO:0003677, GO:0004519, GO:0046872, GO:0043571, GO:0051607, GO:0090305	DNA binding, endonuclease activity, metal ion binding, maintenance of CRISPR repeat elements, defense response to virus, nucleic acid phosphodiester bond hydrolysis
gene_1621	*cas2*	1,790,836–1,791,141	+	306	MULTISPECIES: CRISPR-associated endonuclease Cas2	GO:0004521, GO:0046872, GO:0043571, GO:0051607, GO:0090502	Endoribonuclease activity, metal ion binding, maintenance of CRISPR repeat elements, defense response to virus, RNA phosphodiester bond hydrolysis, endonucleolytic
gene_1622	gene_1622*	1,791,138-1,791,815	+	678	Type II-A CRISPR-associated protein Csn2	−	−
gene_2923	*cas1*	3,233,320–3,234,273	+	954	Subtype I-E CRISPR-associated endonuclease Cas1	GO:0003677, GO:0004519, GO:0046872, GO:0043571, GO:0051607, GO:0090305	DNA binding, endonuclease activity, metal ion binding, maintenance of CRISPR repeat elements, defense response to virus, nucleic acid phosphodiester bond hydrolysis
gene_2924	*FD24_GL002157*	3,234,270–3,235,169	+	900	Type I-E CRISPR-associated endoribonuclease Cas2	GO:0003676	Nucleic acid binding
gene_2925	gene_2925*	3,236,488-3,239,202	+	2,715	CRISPR-associated helicase/endonuclease Cas3	−	−
gene_2926	gene_2926*	3,239,207-3,240,958	+	1752	CRISPR-associated protein	−	−
gene_2927	gene_2927*	3,240,948-3,241,559	+	612	Type I-E CRISPR-associated protein Cse2/CasB	−	−
gene_2928	gene_2928*	3,241,559-3,242,638	+	1,080	Type I-E CRISPR-associated protein Cas7/Cse4/CasC	−	−
gene_2929	*FD24_GL002163*	3,242,619–3,243,344	+	726	Type I-E CRISPR-associated protein Cas5/CasD	GO:0003723, GO:0043571, GO:0051607	RNA binding, maintenance of CRISPR repeat elements, defense response to virus
gene_2930	gene_2930*	3,243,344-3,244,012	+	669	Type I-E CRISPR-associated protein Cas6/Cse3/CasE	−	−

* : New genes found in this study.

**Table 5:** Characterization of CRISPR arrays predicted in the *Lactiplantibacillus pentosus* CF2-10N genome.

CRISPR array (CR)	Start position	End position	Array orientation	CRISPR lenght (bp)	Number of repeats	DR consensus^*^	Array family
CR 1	1,791,840	1,792,537	Forward	698	36	GTCTTGAATAGTAGTCATATCAAACAGGTTTAGAAC	NA
CR 2	2,982,059	2,981,480	Reverse	580	28	CTGTTCCCCGTGTATGCGGGGGTGATCC	I-E
CR 3	3,232,173	3,231,961	Reverse	213	28	CTATTCCCCGTGCATACGGGGGTGATCC	NA
CR 4	3,232,764	3,232,310	Reverse	455	28	CTGTTCCCCGCGTATGCGGGGGTGATCC	I-E
CR 5	3,235,959	3,235,382	Reverse	578	28	CTGTTCCCCGTGTATGCGGGGGTGATCC	I-E

* The same DR consensus sequences are indicated.

### Identification of genes associated with probiotic characteristics in *Lactiplantibacilluis pentosus* CF2-10 N

*In silico* genome analysis of probiotic characteristics of *L. pentosus* CF2-10 N revealed the presence of genes coding for adhesion, exopolysaccharide biosynthesis, tolerance to low pH and bile salts, vitamin and enzyme production and immunomodulation among others (Table 6). With respect to adhesion, several genes were identified such as 3 mucus-binding proteins, 1 fibronectin/fibrinogen-binding protein, 1 Chitin-binding protein (located on pLPE10-1 plasmid), 1 ABC superfamily ATP binding cassette transporter, binding protein, 2 cell surface proteins, 1 manganese ABC transporter substrate-binding protein, 1 elongation factor Tu, 1 Molecular chaperone DnaK, 1 molecular chaperone GroEL, 1 co-chaperone GroES, 1 class A sortase and 1 type I glyceraldehyde-3-phosphate dehydrogenase (Table 6). Regarding exopolysaccharides, four genes coding for exopolysaccharide biosynthesis protein were identified (Table 6). For adaptation to different lifestyles, *L. pentosus* CF2-10 N harbored in its genome several genes involved in stress response such as acids and bile. These included three GNAT family acetyltransferases, two Na+/H+ antiporter NhaC, 1 phosphoglycerate mutase, nine elongation factors (factor G, factor GreA, factor 4, factor P, factor Ts and factor Tu) and 1 phosphoglycerate kinase (Table 6).

**Table 6:** Characterization of genes associated with probiotic properties predicted in the *Lactiplantibacillus pentosus* CF2-10N genome.

Probiotic property		Gene ID	Gene	Position	Strand	Gene length	Protein description	Ontology term (Ontology ID)	COG class (COG class description)
**Adhesion**			gene_411	*FD24_GL003356*	445,136–448,294	−	3,159	Mucus-binding protein	Integral component of membrane (GO:0016021)	COG3846 (Type IV secretory pathway, TrbL components)		
		gene_963	*LPE_00710*	1,054,378–1,060,929	−	6,552	Mucus-binding protein	Integral component of membrane (GO:0016021)	COG0810 (Periplasmic protein TonB, links inner and outer membranes)		
		gene_3039	*gene_3039*	3,352,844–3,359,728	−	6,885	Mucus-binding protein	−	COG5099 (RNA-binding protein of the Puf family, translational repressor)		
		gene_3173	*gene_3173*	3,497,668–3,499,374	−	1707	Fibronectin/fibrinogen-binding protein	−	COG1293 (Predicted RNA-binding protein homologous to eukaryotic snRNP)		
		gene_3512^£^	*gene_3512*	78,678-78,812	+	135	Chitin-binding protein	−	COG3397 (Uncharacterized protein conserved in bacteria)		
		gene_891	*LPE_02200*	975,971–976,864	−	894	ABC superfamily ATP binding cassette transporter, binding protein	Metal ion binding, cell adhesion, metal ion transport (GO:0046872, GO:0007155, GO:0030001)	COG0803 (ABC-type metal ion transport system, periplasmic component/surface adhesin)		
		gene_517	*LPE_00567*	561,619–563,421	+	1803	Cell surface protein	Extracellular region, cell wall, integral component of membrane, collagen binding, cell adhesion (GO:0005576, GO:0005618, GO:0016021, GO:0005518, GO:0007155)	COG0810 (Periplasmic protein TonB, links inner and outer membranes)		
		gene_840	*FD24_GL000462*	920,457–922,340	−	1884	Cell surface protein	Extracellular region, cell wall, collagen binding, cell adhesion (GO:0005576, GO:0005618, GO:0005518, GO:0007155)	COG4932 (Predicted outer membrane protein)		
		gene_2496	*FD24_GL000106*	2,735,640–2,736,581	+	942	Manganese ABC transporter substrate-binding protein	Metal ion binding, cell adhesion, metal ion transport (GO:0005576, GO:0005618, GO:0005518, GO:0007155)	COG0803 (ABC-type metal ion transport system, periplasmic component/surface adhesin)		
		gene_158	*tuf*	162,869–164,056	−	1,188	Elongation factor Tu	Cytoplasm, translation elongation factor activity, GTPase activity, GTP binding, translational elongation (GO:0005737, GO:0003746, GO:0003924, GO:0005525, GO:0006414)	COG0050 (GTPases, translation elongation factors)		
		gene_74	*dnaK*	74,096–75,964	−	1869	Molecular chaperone DnaK	ATP binding,unfolded protein binding,protein folding (GO:0005524, GO:0051082, GO:0006457)	COG0443 (Molecular chaperone)		
		gene_2181	*groL*	2,382,568–2,384,193	+	1,626	MULTISPECIES: molecular chaperone GroEL	Cytoplasm, ATP binding, unfolded protein binding, protein refolding (GO:0005737, GO:0005524, GO:0051082, GO:0042026)	COG0459 [Chaperonin GroEL (HSP60 family)]		
		gene_2180	*groS*	2,382,228–2,382,512	+	285	MULTISPECIES: co-chaperone GroES	cytoplasm, ATP binding, protein folding (GO:0005737, GO:0005524, GO:0006457)	COG0234 [Co-chaperonin GroES (HSP10)]		
		gene_1964	*N692_13295*	2,164,842–2,165,546	+	705	MULTISPECIES: class A sortase	Integral component of membrane (GO:0016021)	COG3764 [Sortase (surface protein transpeptidase)]		
		gene_2239	*LPENT_01088*	2,455,749–2,456,771	+	1,023	MULTISPECIES: type I glyceraldehyde-3-phosphate dehydrogenase	Glyceraldehyde-3-phosphate dehydrogenase (NAD+) (phosphorylating) activity, NADP binding, NAD binding, glucose metabolic process, oxidation–reduction process (GO:0004365, GO:0050661, GO:0051287, GO:0006006, GO:0055114)	COG0057 (Glyceraldehyde-3-phosphate dehydrogenase/erythrose-4-phosphate dehydrogenase)
**Exopolysaccharides**			gene_146	*LPE_00040*	151,375–152,091	−	717	Exopolysaccharide biosynthesis protein	Extracellular polysaccharide biosynthetic process (GO:0045226)	COG0489 (ATPases involved in chromosome partitioning)		
		gene_2641	*LPE_02641*	2,877,168–2,877,944	+	777	Exopolysaccharide biosynthesis protein	Transferase activity, transferring glycosyl groups (GO:0016757)	-		
		gene_2651	*LPE_00805*	2,887,199–2,887,927	+	729	Exopolysaccharide biosynthesis protein	Extracellular polysaccharide biosynthetic process (GO:0045226)	COG0489 (ATPases involved in chromosome partitioning)		
		gene_2,676	*LPE_00838*	2,913,577–2,914,353	+	777	Exopolysaccharide biosynthesis protein	Transferase activity, transferring glycosyl groups (GO:0016757)	-
**Tolerance to low pH and bile salts**			gene_74	*dnaK*	74,096–75,964	−	1869	Molecular chaperone DnaK	ATP binding,unfolded protein binding,protein folding (GO:0005524, GO:0051082, GO:0006457)	COG0443 (Molecular chaperone)		
		gene_607	*pyrD*	664,092–665,009	−	918	Dihydroorotate dehydrogenase B catalytic subunit	Cytoplasm, dihydroorotate dehydrogenase activity, ‘*de novo*’ pyrimidine nucleobase biosynthetic process, ‘*de novo*’ UMP biosynthetic process, oxidation–reduction process (GO:0005737, GO:0004152, GO:0006207, GO:0044205, GO:0055114)	COG0167 (Dihydroorotate dehydrogenase)		
		gene_668	*LPE_01537*	724,936–726,117	+	1,182	GNAT family acetyltransferase	N-acetyltransferase activity (GO:0008080)	COG4552 (Predicted acetyltransferase involved in intracellular survival and related acetyltransferases)		
		gene_1189	*LPE_01193*	1,299,104–1,299,562	+	459	GNAT family acetyltransferase	N-acetyltransferase activity (GO:0008080)	COG2153 (Predicted acyltransferase)		
		gene_1799	*LPE_00911*	1,991,014–1,991,502	−	489	GNAT family acetyltransferase	N-acetyltransferase activity (GO:0008080)	COG2153 (Predicted acyltransferase)		
		gene_1172	*FD24_GL001267*	1,284,763–1,286,187	−	1,425	Na+/H+ antiporter NhaC	Integral component of membrane, antiporter activity, transmembrane transport (GO:0016021, GO:0015297, GO:0055085)	COG1757 (Na+/H+ antiporter)		
		gene_1684	*LPE_02128*	1,859,459–1,860,859	−	1,401	Na+/H+ antiporter NhaC	Integral component of membrane, antiporter activity, transmembrane transport (GO:0016021, GO:0015297, GO:0055085)	COG1757 (Na+/H+ antiporter)		
		gene_2117	*gpmA*	2,321,232–2,321,909	+	678	Phosphoglycerate mutase	2,3-bisphosphoglycerate-dependent phosphoglycerate mutase activity, gluconeogenesis, glycolytic process (GO:0046538, GO:0006094, GO:0006096)	COG0588 (Phosphoglycerate mutase 1)		
		gene_2181	*groL*	2,382,568–2,384,193	+	1,626	MULTISPECIES: molecular chaperone GroEL	Cytoplasm, ATP binding, unfolded protein binding, protein refolding (GO:0005737, GO:0005524, GO:0051082, GO:0042026)	COG0459 (Chaperonin GroEL (HSP60 family)		
		gene_2225	*luxS*	2,438,807–2,439,283	+	477	MULTISPECIES: S-ribosylhomocysteine lyase	Iron ion binding, S-ribosylhomocysteine lyase activity, quorum sensing (GO:0005506, GO:0043768, GO:0009372)	COG1854 (LuxS protein involved in autoinducer AI2 synthesis)		
		gene_2436	*fusA*	2,685,279–2,687,375	+	2097	MULTISPECIES: elongation factor G	Cytoplasm, translation elongation factor activity, GTPase activity, GTP binding, translational elongation (GO:0005737, GO:0003746, GO:0003924, GO:0005525, GO:0006414)	COG0480 [(Translation elongation factors (GTPases)]		
		gene_2966	*greA2*	3,282,534–3,283,016	+	483	MULTISPECIES: transcription elongation factor GreA	DNA binding, translation elongation factor activity, RNA polymerase binding, transcription, DNA-templated, translational elongation, regulation of DNA-templated transcription, elongation (GO:0003677, GO:0003746, GO:0070063, GO:0006351, GO:0006414, GO:0032784)	COG0782 (Transcription elongation factor)		
		gene_1712	*greA*	1,893,369–1,893,839	−	471	Transcription elongation factor GreA	DNA binding, translation elongation factor activity, RNA polymerase binding, transcription, DNA-templated, translational elongation, regulation of DNA-templated transcription, elongation (GO:0003677, GO:0003746, GO:0070063, GO:0006351, GO:0006414, GO:0032784)	COG0782 (Transcription elongation factor)		
		gene_2240	*pgk*	2,456,889–2,458,091	+	1,203	MULTISPECIES: phosphoglycerate kinase	Cytoplasm, phosphoglycerate kinase activity, ATP binding, glycolytic process (GO:0005737, GO:0004618, GO:0005524, GO:0006096)	COG0126 (3-phosphoglycerate kinase)		
		gene_66	*lepA*	64,623–66,458	−	1836	Elongation factor 4	Plasma membrane, translation elongation factor activity, GTPase activity, GTP binding, ribosome binding, translational elongation, positive regulation of translation (GO:0005886, GO:0003746, GO:0003924, GO:0005525, GO:0043022, GO:0006414, GO:0045727)	COG0481 (Membrane GTPase LepA)		
		gene_1072	*lepA*	1,171,570–1,173,357	+	1788	Elongation factor 4	Plasma membrane, translation elongation factor activity, GTPase activity, GTP binding, ribosome binding, translational elongation, positive regulation of translation (GO:0005886, GO:0003746, GO:0003924, GO:0005525, GO:0043022, GO:0006414, GO:0045727)	COG0481 (Membrane GTPase LepA)		
		gene_1569	*FD24_GL002972*	1,732,533–1,734,524	+	1992	Elongation factor G	Translation elongation factor activity, GTPase activity, GTP binding, translational elongation (GO:0003746, GO:0003924, GO:0005525, GO:0006414)	COG0480 [Translation elongation factors (GTPases)]		
		gene_2996	*efp*	3,308,149–3,308,706	+	558	MULTISPECIES: elongation factor P	Cytoplasm, translation elongation factor activity, translational elongation (GO:0005737, GO:0003746, GO:0006414)	COG0231 [(Translation elongation factor P (EF-P)/translation initiation factor 5A (eIF-5A)]		
		gene_101	*tsf*	107,593–108,471	−	879	MULTISPECIES: elongation factor Ts	Cytoplasm, translation elongation factor activity, translational elongation (GO:0005737, GO:0003746, GO:0006414)	COG0264 (Translation elongation factor Ts)		
		gene_158	*tuf*	162,869–164,056	−	1,188	Elongation factor Tu	Cytoplasm, translation elongation factor activity, GTPase activity, GTP binding, translational elongation (GO:0005737, GO:0003746, GO:0003924, GO:0005525, GO:0006414)	COG0050 (GTPases, translation elongation factors)
**Enzymes**			gene_11	*gene_11*	7,804–9,687	−	1884	Tannase	−	−		
		gene_3293	*gene_3293*	3,633,861–3,635,744	−	1884	Tannase	−	−		
		gene_1672	*FD24_GL003074*	1,841,220–1,842,542	+	1,323	Alpha-amylase	Alpha-amylase activity, carbohydrate metabolic process (GO:0004556, GO:0005975)	COG0366 (Glycosidases)		
		gene_1516	*LPE_01041*	1,679,552–1,681,369	+	1818	Amylopullulanase	Alpha-amylase activity,carbohydrate metabolic process (GO:0004556, GO:0005975)	COG0366 (Glycosidases)		
		gene_1271	*FD24_GL001081*	1,379,908–1,381,959	−	2052	Beta-galactosidase	Beta-galactosidase complex, beta-galactosidase activity, metal ion binding, galactose metabolic process (GO:0009341, GO:0004565, GO:0046872, GO:0006012)	COG1874 (Beta-galactosidase)		
		gene_1284	*FD24_GL001068*	1,394,185–1,396,065	+	1881	Beta-galactosidase	Hydrolase activity, hydrolyzing O-glycosyl compounds, carbohydrate metabolic process (GO:0004553, GO:0005975)	COG3250 (Beta-galactosidase/beta-glucuronidase)		
		gene_1285	*FD24_GL001067*	1,396,049–1,397,008	+	960	Beta-galactosidase	Beta-galactosidase complex, beta-galactosidase activity, carbohydrate binding, carbohydrate metabolic process (GO:0009341, GO:0004565, GO:0030246, GO:0005975)	COG3250 (Beta-galactosidase/beta-glucuronidase)		
		gene_1422	*FD24_GL001161*	1,558,889–1,561,432	−	2,544	Hypothetical protein	beta-galactosidase complex, beta-galactosidase activity, carbohydrate metabolic process (GO:0009341, GO:0004565, GO:0005975)	COG1874 (Beta-galactosidase)		
		gene_1988	*FD24_GL001963*	2,194,635–2,195,540	−	906	MULTISPECIES: prolyl aminopeptidase	Aminopeptidase activity, proteolysis (GO:0004177, GO:0006508)	COG0596 [Predicted hydrolases or acyltransferases (alpha/beta hydrolase superfamily)]		
		gene_1749	*map*	1,926,072–1,926,863	−	792	MULTISPECIES: type I methionyl aminopeptidase	Metal ion binding, metalloaminopeptidase activity, proteolysis, protein initiator methionine removal (GO:0046872, GO:0070006, GO:0006508, GO:0070084)	COG0024 (Methionine aminopeptidase)		
		gene_2295	*FD24_GL002755*	2,525,861–2,526,769	−	909	Prolyl aminopeptidase	Aminopeptidase activity, proteolysis (GO:0004177, GO:0006508)	COG0596 [(Predicted hydrolases or acyltransferases (alpha/beta hydrolase superfamily)]		
		gene_1485	*LPE_01265*	1,634,935–1,636,251	−	1,317	Aminopeptidase	Aminopeptidase activity, cysteine-type endopeptidase activity, proteolysis (GO:0004177, GO:0004197, GO:0006508)	COG3579 (Aminopeptidase C)		
		gene_2120	*LPENT_01205*	2,325,277–2,326,608	−	1,332	Aminopeptidase	Aminopeptidase activity, cysteine-type endopeptidase activity, proteolysis (GO:0004177, GO:0004197, GO:0006508)	COG3579 (Aminopeptidase C)		
		gene_2366	*FD24_GL000247*	2,600,983–2,603,517	+	2,535	Peptidase	aminopeptidase activity, metallopeptidase activity, zinc ion binding, proteolysis (GO:0004177, GO:0008237, GO:0008270, GO:0006508)	COG0308 (Aminopeptidase N)		
		gene_281	*pepQ*	299,603–300,712	+	1,110	Peptidase M24 family protein	Hydrolase activity (GO:0016787)	COG0006 (Xaa-Pro aminopeptidase)		
		gene_2995	*LPE_00442*	3,307,014–3,308,078	+	1,065	Peptidase M24 family protein	Aminopeptidase activity, metal ion binding, proteolysis (GO:0004177, GO:0046872, GO:0006508)	COG0006 (Xaa-Pro aminopeptidase)		
		gene_3265	*pepT*	3,600,534–3,601,772	−	1,239	Peptidase T	Cytoplasm, metallopeptidase activity, zinc ion binding, tripeptide aminopeptidase activity, proteolysis, peptide catabolic process (GO:0005737, GO:0008237, GO:0008270, GO:0045148, GO:0006508, GO:0043171)	COG2195 (Di- and tripeptidases)		
		gene_3297	*LPE_00163*	3,638,228–3,639,118	−	891	Alpha/beta hydrolase	serine-type peptidase activity, proteolysis (GO:0008236, GO:0006508)	COG1506 (Dipeptidyl aminopeptidases/acylaminoacyl-peptidases)		
		gene_15	*LPE_00163*	12,171–13,061	−	891	Alpha/beta hydrolase	serine-type peptidase activity,proteolysis	COG1506 (Dipeptidyl aminopeptidases/acylaminoacyl-peptidases)		
		gene_1479	*FD24_GL000907*	1,630,300–1,630,836	+	537	Phenolic acid decarboxylase	carboxy-lyase activity (GO:0016831)	COG3479 (Phenolic acid decarboxylase)		
		ene_2246	*LPE_03197*	2,463,498–2,464,244	+	747	Carboxylesterase	Carboxylic ester hydrolase activity (GO:0052689)	COG1647 (Esterase/lipase)		
		gene_77	*FD24_GL001463*	78,101–78,811	−	711	Alpha-acetolactate decarboxylase	Acetolactate decarboxylase activity, acetoin biosynthetic process (GO:0047605, GO:0045151)	COG3527 (Alpha-acetolactate decarboxylase)		
		gene_808	*FD24_GL001032*	883,614–884,444	−	831	Lipase esterase	Hydrolase activity,metabolic process (GO:0016787,GO:0008152)	COG0657 (Esterase/lipase)		
		gene_1852	*LPE_00868*	2,040,108–2,041,613	+	1,506	MULTISPECIES: multicopper oxidase	Copper ion binding, oxidoreductase activity, cell division, oxidation–reduction process (GO:0005507, GO:0016491, GO:0051301, GO:0055114)	COG2132 (Putative multicopper oxidases)
**Vitamins**	**Follate**	gene_335	*FD24_GL000368*	362,374–363,699	−	1,326	Bifunctional folylpolyglutamate synthase/dihydrofolate synthase	Tetrahydrofolylpolyglutamate synthase activity, ATP binding, tetrahydrofolylpolyglutamate biosynthetic process (GO:0004326, GO:0005524, GO:0046901)	COG0285 (Folylpolyglutamate synthase)		
		gene_1,140	*LPE_01427*	1,241,881–1,243,029	−	1,149	Dihydropteroate synthase	Dihydropteroate synthase activity, folic acid-containing compound biosynthetic process (GO:0004156, GO:0009396)	COG0294 (Dihydropteroate synthase and related enzymes)		
		gene_1145	*LPENT_02091*	1,246,075–1,246,443	−	369	Dihydroneopterin aldolase	Dihydroneopterin aldolase activity, tetrahydrofolate biosynthetic process, folic acid biosynthetic process (GO:0004150, GO:0046654, GO:0046656)	COG1539 (Dihydroneopterin aldolase)		
		gene_3158	*fhs*	3,480,634–3,482,289	+	1,656	Formate--tetrahydrofolate ligase	Formate-tetrahydrofolate ligase activity, ATP binding, folic acid-containing compound biosynthetic process, tetrahydrofolate interconversion (GO:0004329, GO:0005524, GO:0009396, GO:0035999)	COG2759 (Formyltetrahydrofolate synthetase)		
		gene_1143	*folE*	1,245,011–1,245,580	−	570	MULTISPECIES: GTP cyclohydrolase I FolE	Cytoplasm, GTP cyclohydrolase I activity, GTP binding, zinc ion binding, one-carbon metabolic process,7,8-dihydroneopterin 3′-triphosphate biosynthetic process, tetrahydrofolate biosynthetic process (GO:0005737, GO:0003934, GO:0005525, GO:0008270, GO:0006730, GO:0035998, GO:0046654)	COG0302 (GTP cyclohydrolase I)		
		gene_1144	*LPE_01431*	1,245,573–1,246,085	−	513	2-amino-4-hydroxy-6-hydroxymethyldihydropteridine diphosphokinase	2-amino-4-hydroxy-6-hydroxymethyldihydropteridine diphosphokinase activity, kinase activity, folic acid-containing compound biosynthetic process, phosphorylation (GO:0003848, GO:0016301, GO:0009396, GO:0016310)	COG0801 (7,8-dihydro-6-hydroxymethylpterin-pyrophosphokinase)		
		gene_2999	*folD*	3,309,775–3,310,635	+	861	Bifunctional protein folD	Methenyltetrahydrofolate cyclohydrolase activity, methylenetetrahydrofolate dehydrogenase (NADP+) activity, histidine biosynthetic process, purine nucleotide biosynthetic process, methionine biosynthetic process, folic acid-containing compound biosynthetic process, tetrahydrofolate interconversion, oxidation–reduction process (GO:0004477, GO:0004488, GO:0000105, GO:0006164, GO:0009086, GO:0009396, GO:0035999, GO:0055114)	COG0190 (5,10-methylene-tetrahydrofolate dehydrogenase/Methenyl tetrahydrofolate cyclohydrolase)	
	**Riboflavin**	gene_2293	*LPE_03224*	2,522,788–2,523,636	+	849	Bifunctional protein: riboflavin kinas	FMN adenylyltransferase activity, kinase activity, riboflavin biosynthetic process, phosphorylation (GO:0003919, GO:0016301, GO:0009231, GO:0016310)	COG0196 (FAD synthase)		
		gene_78	*FD24_GL001464*	78,815–79,813	−	999	Bifunctional riboflavin kinase/FMN adenylyltransferase	FMN adenylyltransferase activity, ATP binding, riboflavin kinase activity, FAD biosynthetic process, riboflavin biosynthetic process, FMN biosynthetic process, phosphorylation (GO:0003919, GO:0005524, GO:0008531, GO:0006747, GO:0009231, GO:0009398, GO:0016310)	COG0196 (FAD synthase)		
		gene_2838	*FD24_GL002070*	3,139,751–3,140,962	+	1,212	bifunctional 3,4-dihydroxy-2-butanone-4-phosphate synthase/GTP cyclohydrolase II	GTP cyclohydrolase II activity, GTP binding, 3,4-dihydroxy-2-butanone-4-phosphate synthase activity, metal ion binding, riboflavin biosynthetic process (GO:0003935, GO:0005525, GO:0008686, GO:0046872, GO:0009231)	COG0807 (GTP cyclohydrolase II)		
		gene_2839	*ribH*	3,140,962–3,141,429	+	468	6,7-dimethyl-8-ribityllumazine synthase	riboflavin synthase complex,6,7-dimethyl-8-ribityllumazine synthase activity, transferase activity, riboflavin biosynthetic process (GO:0009349, GO:0000906, GO:0016740, GO:0009231)	COG0054 (Riboflavin synthase beta-chain)		
		gene_2836	*LPE_03075*	3,138,079–3,139,146	+	1,068	Riboflavin biosynthesis protein RibD	Zinc ion binding,5-amino-6-(5-phosphoribosylamino) uracil reductase activity, diaminohydroxyphosphoribosylaminopyrimidine deaminase activity, riboflavin biosynthetic process, oxidation–reduction process (GO:0008270, GO:0008703, GO:0008835, GO:0009231, GO:0055114)	COG1985 (Pyrimidine reductase, riboflavin biosynthesis)		
		gene_3254	*gene_3254*	3,584,670–3,585,050	−	381	Riboflavin biosynthesis protein RibT	−	−		
		gene_2837	*LPE_03076*	3,139,147–3,139,749	+	603	Riboflavin synthase	Oxidoreductase activity,oxidation–reduction process (GO:0016491, GO:0055114)	COG0307 (Riboflavin synthase alpha chain)		
		gene_728	*LPENT_02492*	795,860–796,399	+	540	Dihydrofolate reductase	integral component of membrane,5-amino-6-(5-phosphoribosylamino) uracil reductase activity, riboflavin biosynthetic process, oxidation–reduction process (GO:0016021, GO:0008703, GO:0009231, GO:0055114)	COG0262 (Dihydrofolate reductase)	
	**Thiamine**	gene_1606	*thiE*	1,772,372–1,773,028	+	657	Thiamine phosphate synthase	Magnesium ion binding, thiamine-phosphate diphosphorylase activity, thiamine biosynthetic process, thiamine diphosphate biosynthetic process (GO:0000287, GO:0004789, GO:0009228, GO:0009229)	COG0352 (Thiamine monophosphate synthase)		
		gene_532	*LPE_00578*	575,356–577,104	−	1749	1-deoxy-D-xylulose-5-phosphate synthase	1-deoxy-D-xylulose-5-phosphate synthase activity, metal ion binding, thiamine biosynthetic process, terpenoid biosynthetic process, 1-deoxy-D-xylulose 5-phosphate biosynthetic process (GO:0008661, GO:0046872, GO:0009228, GO:0016114, GO:0052865)	COG1154 (Deoxyxylulose-5-phosphate synthase)		
		gene_1604	*thiM*	1,770,746–1,771,540	+	795	Hydroxyethylthiazole kinase	Magnesium ion binding, hydroxyethylthiazole kinase activity, ATP binding, thiamine biosynthetic process, thiamine diphosphate biosynthetic process, phosphorylation (GO:0000287, GO:0004417, GO:0005524, GO:0009228, GO:0009229, GO:0016310)	COG2145 (Hydroxyethylthiazole kinase, sugar kinase family)		
		gene_2902	*FD24_GL002133*	3,205,215–3,206,249	−	1,035	Molybdopterin biosynthesis protein MoeB	Small protein activating enzyme activity (GO:0008641)	COG0476 (Dinucleotide-utilizing enzymes involved in molybdopterin and thiamine biosynthesis family 2)		
		gene_1605	*FD24_GL003009*	1,771,558–1,772,382	+	825	MULTISPECIES: hydroxymethylpyrimidine/phosphomethylpyrimidine kinase	ATP binding, phosphomethylpyrimidine kinase activity, thiamine biosynthetic process, phosphorylation (GO:0005524, GO:0008972, GO:0009228, GO:0016310)	COG0351 (Hydroxymethylpyrimidine/phosphomethylpyrimidine kinase)		
		gene_3021	*LPE_00414*	3,332,290–3,332,946	+	657	Thiamine pyrophosphokinase	thiamine diphosphokinase activity, ATP binding, thiamine binding, thiamine metabolic process, thiamine diphosphate biosynthetic process (GO:0004788, GO:0005524, GO:0030975, GO:0006772, GO:0009229)	COG1564 (Thiamine pyrophosphokinase)		
		gene_339	*thiI*	369,546–370,763	−	1,218	tRNA sulfurtransferase	cytoplasm, tRNA binding, tRNA adenylyltransferase activity,ATP binding, sulfurtransferase activity, thiamine biosynthetic process, thiamine diphosphate biosynthetic process, tRNA thio-modification (GO:0005737, GO:0000049, GO:0004810, GO:0005524, GO:0016783, GO:0009228, GO:0009229, GO:0034227)	COG0301 (Thiamine biosynthesis ATP pyrophosphatase)		
		gene_821	*FD24_GL000441*	897,704–898,705	+	1,002	FAD:protein FMN transferase	Transferase activity, metal ion binding, protein flavinylation (GO:0016740, GO:0046872, GO:0017013)	COG1477 (Membrane-associated lipoprotein involved in thiamine biosynthesis)		
		gene_1301	*LPE_02537*	1,421,909–1,422,865	−	957	FAD:protein FMN transferase	Transferase activity, metal ion binding, protein flavinylation (GO:0016740, GO:0046872, GO:0017013)	COG1477 (Membrane-associated lipoprotein involved in thiamine biosynthesis)		
		gene_2469	*FD24_GL000140*	2,709,017–2,710,129	+	1,113	FAD:protein FMN transferase	Transferase activity, metal ion binding, protein flavinylation (GO:0016740, GO:0046872, GO:0017013)	COG1477 (Membrane-associated lipoprotein involved in thiamine biosynthesis)	
	**Vitamin K2**	gene_1240	*menG*	1,346,872–1,347,585	+	714	Bifunctional demethylmenaquinone methyltransferase/2-methoxy-6-polyprenyl-1,4-benzoquinol methylase	Methyltransferase activity, menaquinone biosynthetic process, methylation (GO:0008168, GO:0009234, GO:0032259)	COG2226 (Methylase involved in ubiquinone/menaquinone biosynthesis)	
	**Vitamin B5**	gene_452	*hdhD1*	486,520–487,494	−	975	2-dehydropantoate 2-reductase	Cytoplasm, 2-dehydropantoate 2-reductase activity, NADP binding, pantothenate biosynthetic process, oxidation–reduction process (GO:0005737, GO:0008677, GO:0050661, GO:0015940, GO:0055114)	COG1893 (Ketopantoate reductase)		
		gene_693	*gene_693*	750,504–751,523	+	1,020	2-dehydropantoate 2-reductase	−	COG1893 (Ketopantoate reductase)		
		gene_1840	*LPE_00879*	2,026,749–2,027,732	+	984	2-dehydropantoate 2-reductase	Cytoplasm, 2-dehydropantoate 2-reductase activity, NADP binding, pantothenate biosynthetic process, oxidation–reduction process (GO:0005737, GO:0008677, GO:0050661, GO:0015940, GO:0055114)	COG1893 (Ketopantoate reductase)
									
	**Vitamin B6**	gene_1521	*FD24_GL000863*	1,687,267–1,687,710	−	444	MULTISPECIES: pyridoxamine 5′-phosphate oxidase	Pyridoxamine-phosphate oxidase activity, FMN binding, pyridoxal phosphate biosynthetic process, oxidation–reduction process (GO:0004733, GO:0010181, GO:0042823, GO:0055114)	-
			gene_653	*FD24_GL002535*	711,251–712,435	−	1,185	Pyridoxal phosphate-dependent aminotransferase	Transaminase activity, pyridoxal phosphate binding, biosynthetic process (GO:0008483, GO:0030170, GO:0009058)	COG1168 (Bifunctional PLP-dependent enzyme with beta-cystathionase and maltose regulon repressor activities)
			gene_780	*LPE_03241*	846,681–847,853	−	1,173	Pyridoxal phosphate-dependent aminotransferase	transaminase activity, pyridoxal phosphate binding, biosynthetic process (GO:0008483, GO:0030170, GO:0009058)	COG1168 (Bifunctional PLP-dependent enzyme with beta-cystathionase and maltose regulon repressor activities)
			gene_1841	*LPE_00878*	2,027,735–2,028,907	+	1,173	Pyridoxal phosphate-dependent aminotransferase	transaminase activity,pyridoxal phosphate binding,biosynthetic process (GO:0008483, GO:0030170, GO:0009058)	COG0436 (Aspartate/tyrosine/aromatic aminotransferase)
			gene_3128	*LPE_00325*	3,447,981–3,449,180	+		Pyridoxal phosphate-dependent aminotransferase	L-aspartate:2-oxoglutarate aminotransferase activity,pyridoxal phosphate binding,L-phenylalanine:2-oxoglutarate aminotransferase activity,biosynthetic process (GO:0004069, GO:0030170,GO:0080130, GO:0009058)	COG0436 (Aspartate/tyrosine/aromatic aminotransferase)
			gene_2303	*LPE_03213*	2,538,192–2,539,010	+		Pyridoxine kinase	ATP binding,pyridoxal kinase activity,pyridoxal 5′-phosphate salvage,phosphorylation (GO:0005524, GO:0008478, GO:0009443, GO:0016310)	COG2240 (Pyridoxal/pyridoxine/pyridoxamine kinase)

* : the best hit was indicated.

£ : sequences of pLPE10-1 plasmid.

§ : sequences of pLPE10-4 plasmid.

& : sequences of pLPE10-2 plasmid.

On the other hand, several genes were identified coding for enzymes involved in probiotic functions such as two genes coding for tannase (exclusive to this strain), 1 alpha-amylase, 1 amylopullulanase, 3 beta-galactosidases, 5 aminopeptidases, 1 lipase esterase, 4 peptidases, 2 alpha/beta hydrolases, 1 phenolic acid decarboxylase, 1 carboxylesterase, 1 alpha-acetolactate decarboxylase, and 1 multicopper oxidase (Table 6).

With respect to vitamin biosynthesis, we detected genes coding for proteins involved in vitamins B1 or thiamine (10 genes), B2 or riboflavin (8 genes), B5 (3 genes) and B6 (6 genes), folate (7 genes) and vitamin K2 or menaquinone (1 gene) production (Table 6). In this regard, vitamin production ability of *L. pentosus* CF2-10 N was validated *in vitro*.

## Discussion

Aloreña table olives, naturally fermented traditional green olives with a denomination of protection (DOP), are considered as potential source of probiotic *L. pentosus* strains with high genetic diversity (Abriouel et al., 2012). Several *L. pentosus* strains isolated from Aloreña table olives throughout the fermentation process were shown to be potential probiotics, with *L. pentosus* MP-10, *L. pentosus* CF1-6 and *L. pentosus* CF2-10 N as the best candidates (Pérez Montoro et al., 2016). Among these strains, *L. pentosus* CF2-10 N was selected for a more in-depth analysis in the current study on the basis of its excellent probiotic properties. These include notably good growth capacity and survival under simulated gastro-intestinal conditions (acidic pH of 1.5, up to 4% of bile salts and 5 mM of nitrate), good ability to auto-aggregate and co-aggregate with pathogenic bacteria, adherence to intestinal and vaginal cell lines, antimicrobial activity by means of plantaricins and fermentation of prebiotics and lactose (Pérez Montoro et al., 2016). It is also noteworthy that *L. pentosus* CF2-10 N was isolated from the same ecological niche as the potential previously described probiotic *L. pentosus* MP-10 (Abriouel et al., 2012), hence, they are exposed to the same ecological conditions and pressure (soil, plant and brine) as well as the same progressive changes throughout the production process. It is thus not surprising that their genetic relatedness is further highlighted by shared genetic, functional and probiotic properties although both strains showed different genomic profiles belonging to different clusters or genomic groups (G1 and G2) as reported by Abriouel et al. (2012). In this sense, both strains harbor a single circular chromosome of similar size of 3,698,214 bp (*L. pentosus* MP-10, GC content of 46.32%) and 3,645,747 bp (*L. pentosus* CF2-10 N, GC content of 46.42%) and 4 (*L. pentosus* CF2-10 N, 58–120 kb) to 5 (*L. pentosus* MP-10, 29–46.5 kb) plasmids (Abriouel et al., 2016). This similarity highlights the effect of the ecosystem (soil, plant and brine) on the genetic diversity of microbial communities present in Aloreña table olives.

A comparison with other bacterial strains from table olives showed similarities in genomic size and GC content. These strains included *L. pentosus* IG1 harboring a circular chromosome of 3,687,424 bp (GC content of 44.9%) and 7 plasmids (2.5–125.9 kb; Maldonado-Barragán et al., 2011), *L. pentosus* strains (IG8, IG9, IG10 and IG11) recovered from biofilms on the skin of green table olives with circular chromosome sizes in the range of 3,787,967 to 3,811,295 bp (GC content of 45.9–45.95%) and 6 to 7 plasmids (Calero-Delgado et al., 2019) and *L. pentosus* O17 isolated from brines of treated table olives (*Cerignola* cv.) with a circular chromosome of 3,850,701 bp (GC content of 45.9%; Zotta et al., 2022). This fact indicated their adaptation to a brine-specific lifestyle notably in relation to genes involved in carbohydrate transport and metabolism (307 CDSs and 279 in *L. pentosus* CF2-10 N and MP-10, respectively) and amino acid metabolism (192 CDSs and 173 in *L. pentosus* CF2-10 N and MP-10, respectively), among others. On the other hand, the presence of plasmids in *L. pentosus* isolated from table olives highlight their key role in the fermentation process. In this sense, Abriouel et al. (2019) reported that *L. pentosus* MP-10 plasmids play an important role as metal bioquencher reducing the amount of these potentially toxic elements in humans and animals, food matrices, and in environmental bioremediation.

Duar et al. (2017) reported a high level of niche conservatism within the well-supported phylogenetic groups of the genus *Lactobacillus* (including the recently reclassified genus *Lactoplantibacillus*), with lifestyles ranging from free-living with large genome size to strictly symbiotic or host adapted with small genome size. Considering that the metabolic and physiological properties of *L. pentosus* strains are reflective of their lifestyle, strains isolated from fermented table olives are characterized by their large genome size of 3.6–3.8 Mbp encoding a versatile repertoire of enzymes to utilize a wide spectrum of substrates available in brines. Comparative genomic analysis of both strains isolated from Aloreña table olives - *L. pentosus* MP-10 and *L. pentosus* CF2-10 N- demonstrated their close phylogenetic relation (ED = 0) and a high similarity although some event traits (inversion, insertion or gene rearrangement) occurred, conferring exclusive features to *L. pentosus* CF2-10 N. However, when genomic comparison was done with *L. pentosus* IG1 isolated from the Spanish-Style Green Olive fermentation (different ecological niche than Aloreña table olives), genetic differences (ED = 0.02) were detected which were further increased when compared with *L. pentosus* KCA1 isolated from vagina (ED = 0.08). The ecological adaptability of *L. pentosus* is thus highly dependent on the ecological niche, with the specific environmental and fermentation conditions and olive material being the key elements to determine the genetic diversity.

Concerning the safety properties of *L. pentosus* CF2-10 N, no ARGs were detected in the genome sequence, however non-specific antimicrobial mechanisms such as mutation in *ddl* gene coding for D-Ala-D-lactate in the peptidoglycan instead of the normal dipeptide D-Ala-D-Ala (position 260) and /or efflux transporters or transmembrane proteins were found responsible of the strain’s phenotypic resistance to streptomycin and vancomycin as detected by antibiotic susceptibility testing (Casado Muñoz et al., 2014). Furthermore, *in silico* analysis of antibiotic resistance in *L. pentosus* CF2-10 N showed the absence of acquired antibiotic resistance genes. Thus, we can conclude that the resistome is mostly represented by efflux-pump resistance genes or other alternative resistance mechanisms responsible for the intrinsic resistance exhibited by this strain as mentioned above. On the other hand, no virulence determinants were detected in the *L. pentosus* CF2-10 N genome. Taken together these results, we suggest for *L. pentosus* CF2-10 N to be considered as safe for food processing as well as probiotic.

Regarding the mobilome (corresponding to genetic elements able to move within a genome or between different genomes), this consists of 66 transposases, 45 IS elements and 8 temperate phage regions in the *L. pentosus* CF2-10 N genome. The high number and the great diversity of transposases and IS elements identified by *in silico* analysis of the *L. pentosus* CF2-10 N genome indicated a frequent genetic diversification within the *L. pentosus* CF2-10 N genome, which is notably higher than in other lactobacilli such as *L. plantarum* WCFS1 (36 genes), *L. pentosus* KCA1 (25 genes), *L. pentosus* DSM 20314 (14 genes) or *L. pentosus* IG1 (5 genes; Abriouel et al., 2017). Interestingly, *L. pentosus* CF2-10 N showed an even higher genetic diversification in comparison to *L. pentosus* MP-10 (29 genes), even though both strains are isolated from the same ecological niche (Abriouel et al., 2017). Furthermore, most of transposases belonged to IS30 families frequently located on plasmids, while the IS were mainly represented by IS30 and IS3 found in various bacteria and being responsible for information transfer and extreme adaptation. This fact suggests the high adaptability potential of *L. pentosus* CF2-10 N enabling the bacterium to withstand different environmental and gut stress conditions. Furthermore, the presence of eight prophage regions in the *L. pentosus* CF2-10 N genome highlights once more the genetic diversity and fitness of its genome, conferring a selective advantage for the survivability and resistance of this strain in view of the potential risk of losses associated with phage infection in different ecosystems. The presence of prophages in lactobacilli genomes is widely distributed (more than 92%, Sun et al., 2015) and is species-specific (Pei et al., 2021), while being highly dependent on the habitat. In this regard *L. pentosus* CF2-10 N contained intact lactobacilli prophage and incomplete or questionable prophage fragments similar to other bacteria (*Staphylococcus, Escherichia* and Enterobacteria phages) indicating its adaptability to harsh conditions (fermentation) which may confer flexibility against various stress triggers (phages from different sources such as air, water or soil). Other defense mechanisms were predicted in the *L. pentosus* CF2-10 N including a CRISPR system (CRISPR-I and CRISPR-II) represented by five CRISPR unquestionable arrays and 13 CRISPR associated proteins (six of them were exclusive of this strain) organized in two operons. This acquired immunity system, which provides protection against mobile genetic elements (conjugative plasmids, transposable elements, and phages) in *L. pentosus* CF2-10 N, was slightly different from *L. pentosus* MP-10 isolated from the same ecological niche. Notably, 11 CRISPR associated proteins and 9 CRISPR arrays (3 of them were questionable CRISPRs) were detected in *L. pentosus* MP-10, which indicated that the increased fitness greatly depends on the strain itself, under changing ecological lifestyles. Among the six newly detected genes, the CRISPR-I system was found to be coding for a Type II-A CRISPR-associated protein Csn2, involved in CRISPR adaptation for new spacer acquisition (Nam et al., 2011) and was associated with the *cas9-cas1-cas2* cassette. Furthermore, the other genes (*gene_2925* [*cas 3*] and a cascade of five genes coding for Type I-E CRISPR associated proteins) were found to be involved in interference and infection neutralization as reported by Xue and Sashital (2019).

Concerning functional properties, *L. pentosus* CF2-10 N genome analysis revealed the presence of genes coding for adhesion, exopolysaccharide biosynthesis, tolerance to low pH and bile salts, immunomodulation, as well as vitamin and enzyme production. In this context, the adhesion capacity exhibited by this strain *in vitro* to Enterocyte-like Caco-2 ECACC86010202 (from colon adenocarcinoma) and HeLa 229 ECACC86090201(from vaginal cervix carcinoma) cells (Pérez Montoro et al., 2016) was confirmed by the presence of genes coding for several adhesion/multifunctional proteins such as mucus-binding proteins, fibronectin/fibrinogen-binding protein, Chitin-binding protein, ABC superfamily ATP binding cassette transporter, binding protein, cell surface proteins, manganese ABC transporter substrate-binding protein, elongation factor Tu, Molecular chaperone DnaK, molecular chaperone GroEL, co-chaperone GroES, class A sortase and type I glyceraldehyde-3-phosphate dehydrogenase. These proteins were reported to be involved in the adhesion to intestinal epithelial cells (Granato et al., 2004; Vélez et al., 2007; Lebeer et al., 2008; Sánchez et al., 2011; Jensen et al., 2014; Hymes et al., 2016), however, some of these proteins can also be involved in other functions such as stress response, drug efflux, carbohydrate transport and metabolism and other probiotic actions (Lebeer et al., 2008; Lewis et al., 2012; Monteagudo-Mera et al., 2019). The specific functionality notably depends on the surrounding conditions which induce gene expression, with differences detected in both *in vitro* and *in vivo* scenarios. On the other hand, other genes coding for proteins involved in cell recognition and adhesion to intestinal mucosae such as the four genes coding for exopolysaccharide biosynthesis proteins were identified in the *L. pentosus* CF2-10 N genome. These were found to be identical to those detected in *L. pentosus* MP-10 isolated from Aloreña table olives (Abriouel et al., 2016). Besides their role in niche adaptation, promoting auto-aggregation and biofilm formation, these proteins were also attributed anti-inflammatory, antioxidant, antiviral and antiproliferative activity functions through their interaction with the immune system (Castro-Bravo et al., 2018; Nguyen et al., 2020; Riaz Rajoka et al., 2020).

To allow the adaptation to different lifestyles, *L. pentosus* CF2-10 N harbored in its genome several genes involved in stress response such as acids and bile. In this sense, Pérez Montoro et al. (2016) reported the strain’s excellent tolerance properties *in vitro* (acidic pH of 1.5, up to 4% of bile salts and 5 mM of nitrate), while in the present study we detected for the first time several genes coding for proteins involved in bile/acids resistance particularly including cell protection (*dnaK* and *groL*), modifications in cell membranes (genes coding for Na+/H+ antiporter NhaC, *lepA*, *pyrD*), general function (genes coding for GNAT family acetyltransferase), and key components of central metabolism (*pgk*, *gpm*, CysK, *luxS*, *tuf, efp, tsf, FD24_GL002972*, *greA*, *greA2*, *fusA*) as it was reported elsewhere for other bacteria (Wu et al., 2010; Liu et al., 2018; Bagon et al., 2021). Most of these proteins are considered moonlighting proteins involved in adhesion to the intestinal epithelium among other functions (Pagnini et al., 2018).

Concerning attractive and promising biotechnological features revealed by *in silico* analysis of the *L. pentosus* CF2-10 N genome, detected enzymes were involved in the degradation of toxic/complex substrates such as tannase, alpha-amylase, amylopullulanase, beta-galactosidase, aminopeptidase, lipase esterase, peptidases, alpha/beta hydrolase, phenolic acid decarboxylase, carboxylesterase, alpha-acetolactate decarboxylase and multicopper oxidase. These findings indicate the high adaptability of this strain to a broad range of environmental niches, food matrices and also the gastrointestinal tract, while being able to ferment lactose and starch. Findings further demonstrate the strain’s potential ability to synthesize and degrade a broad array of simple and complex carbohydrates, such as starch, pullulan, amylopectin, tannin, beta-galactosides, phenolic acids and other substrates. It is further noteworthy that *L. pentosus* CF2-10 N harbored genes coding for vitamin biosynthesis such as the vitamin B group (B1 or thiamine, B2 or riboflavin and B5), folate and vitamin K2 or menaquinone. In this regard, preliminary *in vitro* studies hinted towards a potential vitamin production ability of *L. pentosus* CF2-10 N. However, future studies are necessary and will be performed to investigate this potential in further detail.

## Conclusion

The results obtained in the present study support the hypothesis that *L. pentosus* CF2-10 N is an excellent probiotic candidate of vegetable origin. Notably, besides fulfilling the main criteria for probiotic selection *in vitro* as shown by our previous studies, *in silico* genome analysis in this study revealed novel insights into its safety and functionality, greatly highlighting the microorganism’s ecological flexibility and adaptability to a broad range of environmental niches, food matrices and the gastrointestinal tract. The safety of *L. pentosus* CF2-10 N was further confirmed by the absence of virulence determinants and acquired antibiotic resistance genes, with the resistome mostly represented by efflux-pump resistance genes responsible for the intrinsic resistance exhibited by this strain. On the other hand, defense mechanisms of *L. pentosus* CF2-10 N consist of eight prophage regions as well as a CRISPR (clustered regularly interspaced short palindromic repeats)/cas (CRISPR-associated protein genes) system (CRISPR-I and CRISPR-II) as acquired immune system against mobile elements. The latter is notably represented by five CRISPR unquestionable arrays and 13 CRISPR associated proteins (six of them were exclusive of this strain). Furthermore, the functionality of this strain was supported by the presence of genes coding for proteins involved in adhesion, exopolysaccharide biosynthesis, tolerance to low pH and bile salts, immunomodulation as well as vitamin and enzyme production.

Taken together these results we suggest that *L. pentosus* CF2-10 N could be considered as potential and promising probiotic candidate able to colonize several niches and adapt to different lifestyles, while providing attractive probiotic features, which will be explored *in vivo* in future studies with the aim to be applied in vegetable fermentations (including olives) and/or other substrates.

## Data availability statement

The datasets presented in this study can be found in online repositories. The names of the repository/repositories and accession number(s) can be found in the article/Supplementary material.

## Author contributions

HA and NB conceived, designed the experiments, and drafted the paper. HA, JM, NC, and NB performed the experiments and analyzed the data. HA contributed reagents, materials, and analysis tools. All authors contributed to the article and approved the submitted version.

## Conflict of interest

The authors declare that the research was conducted in the absence of any commercial or financial relationships that could be construed as a potential conflict of interest.

## Publisher’s note

All claims expressed in this article are solely those of the authors and do not necessarily represent those of their affiliated organizations, or those of the publisher, the editors and the reviewers. Any product that may be evaluated in this article, or claim that may be made by its manufacturer, is not guaranteed or endorsed by the publisher.

## Footnotes

^1^ http://hmmer.org/

^2^ http://hmmer.org/

^3^ https://cge.cbs.dtu.dk/services/ResFinder/

^4^ http://crispr.otago.ac.nz/CRISPRDetect

^5^ https://card.mcmaster.ca/analyze/rgi
