# Corrigendum: *In silico* genomic analysis of the potential probiotic *Lactiplantibacillus pentosus* CF2-10N reveals promising beneficial effects with health promoting properties

**DOI:** 10.3389/fmicb.2023.1242095

**Published:** 2023-06-28

**Authors:** Hikmate Abriouel, Julia Manetsberger, Natacha Caballero Gómez, Nabil Benomar

**Affiliations:** Área de Microbiología, Departamento de Ciencias de la Salud, Facultad de Ciencias Experimentales, Universidad de Jaén, Jaén, Spain

**Keywords:** Aloreña table olives, *Lactiplantibacillus pentosus*, probiotics, *in silico* analysis, safety, functional properties

In the published article, there was an error in the final paragraph of the **Materials and methods** subsection “Genome assembly and annotation”. The accession number for the complete genome sequence of *L. pentosus* CF2-10N has been corrected from “ERR10150811” to “ERR11550479”.

The authors apologize for this error and state that this does not change the scientific conclusions of the article in any way. The original article has been updated.

