# Supplementary material for: In silico genomic analysis of the potential probiotic Lactiplantibacillus pentosus CF2-10N reveals promising beneficial effects with health promoting properties
Source: Front Microbiol. 2022 Nov 3;13:989824. doi: 10.3389/fmicb.2022.989824 (PMC9670130; doi:10.3389/fmicb.2022.989824)
Supplement: Supplementary file 1 [file Data_Sheet_1.zip › Table S3.docx]

**Table S3.** COG stat of genome features of *Lactiplantibacillus pentosus* CF2-10N.

| **COG* code** | **Function category** | **Number of CDS**** |
| --- | --- | --- |
| C | Energy production and conversion | 107 |
| CE | Multiple classes | 1 |
| CHR | Multiple classes | 3 |
| CO | Multiple classes | 2 |
| CP | Multiple classes | 1 |
| CR | Multiple classes | 10 |
| D | Cell cycle control, cell division, chromosome partitioning | 38 |
| E | Amino acid transport and metabolism | 192 |
| EF | Multiple classes | 6 |
| EH | Multiple classes | 7 |
| EJ | Multiple classes | 1 |
| EM | Multiple classes | 3 |
| EP | Multiple classes | 6 |
| ER | Multiple classes | 11 |
| ET | Multiple classes | 6 |
| F | Nucleotide transport and metabolism | 84 |
| FE | Multiple classes | 2 |
| FGR | Multiple classes | 3 |
| FJ | Multiple classes | 1 |
| FP | Multiple classes | 3 |
| G | Carbohydrate transport and metabolism | 307 |
| GE | Multiple classes | 2 |
| GER | Multiple classes | 6 |
| GM | Multiple classes | 6 |
| GT | Multiple classes | 17 |
| H | Coenzyme transport and metabolism | 89 |
| HE | Multiple classes | 4 |
| HI | Multiple classes | 2 |
| HJ | Multiple classes | 1 |
| I | Lipid transport and metabolism | 59 |
| IQ | Multiple classes | 6 |
| IQR | Multiple classes | 10 |
| IR | Multiple classes | 3 |
| J | Translation, ribosomal structure and biogenesis | 148 |
| K | Transcription | 235 |
| KE | Multiple classes | 3 |
| KG | Multiple classes | 20 |
| KL | Multiple classes | 4 |
| KT | Multiple classes | 14 |
| L | Replication, recombination and repair | 213 |
| LK | Multiple classes | 2 |
| LKJ | Multiple classes | 3 |
| LR | Multiple classes | 2 |
| LU | Multiple classes | 1 |
| M | Cell wall/membrane/envelope biogenesis | 142 |
| MG | Multiple classes | 12 |
| MI | Multiple classes | 2 |
| MR | Multiple classes | 2 |
| MU | Multiple classes | 1 |
| N | Cell motility | 1 |
| NOU | Multiple classes | 1 |
| NU | Multiple classes | 6 |
| O | Posttranslational modification, protein turnover, chaperones | 50 |
| OC | Multiple classes | 2 |
| OTN | Multiple classes | 1 |
| OU | Multiple classes | 3 |
| P | Inorganic ion transport and metabolism | 126 |
| PH | Multiple classes | 2 |
| Q | Secondary metabolites biosynthesis, transport and catabolism | 13 |
| R | General function prediction only | 336 |
| RTKL | Multiple classes | 2 |
| S | Function unknown | 273 |
| T | Signal transduction mechanisms | 70 |
| TK | Multiple classes | 9 |
| TQ | Multiple classes | 3 |
| U | Intracellular trafficking, secretion, and vesicular transport | 28 |
| UW | Multiple classes | 2 |
| V | Defense mechanisms | 70 |

*COG: Clusters of Orthologous Groups

** CDS: Coding Sequence
