# Supplementary figures and images for: In silico genomic analysis of the potential probiotic Lactiplantibacillus pentosus CF2-10N reveals promising beneficial effects with health promoting properties
Source: Front Microbiol. 2022 Nov 3;13:989824. doi: 10.3389/fmicb.2022.989824 (PMC9670130; doi:10.3389/fmicb.2022.989824)

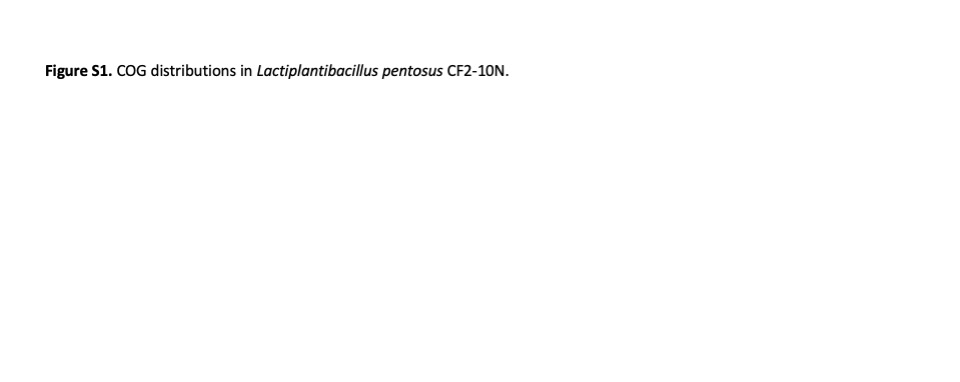

Supplement: Supplementary file 1 [file Data_Sheet_1.zip › Figure S1 caption.JPEG]

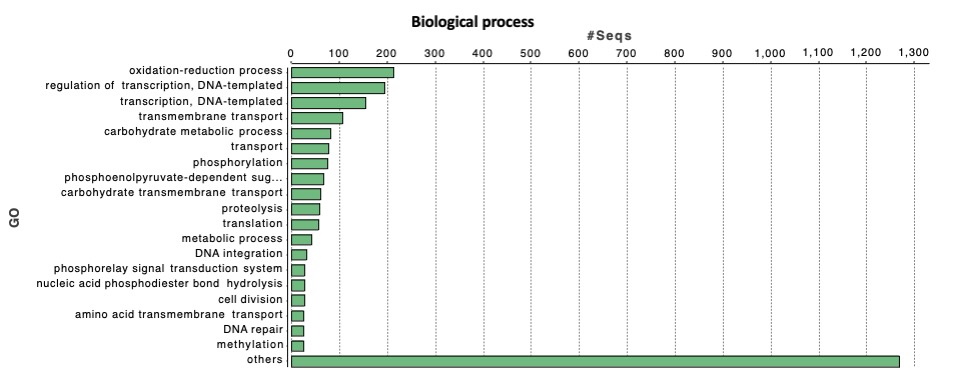

Supplement: Supplementary file 1 [file Data_Sheet_1.zip › Figure S1a.JPEG]

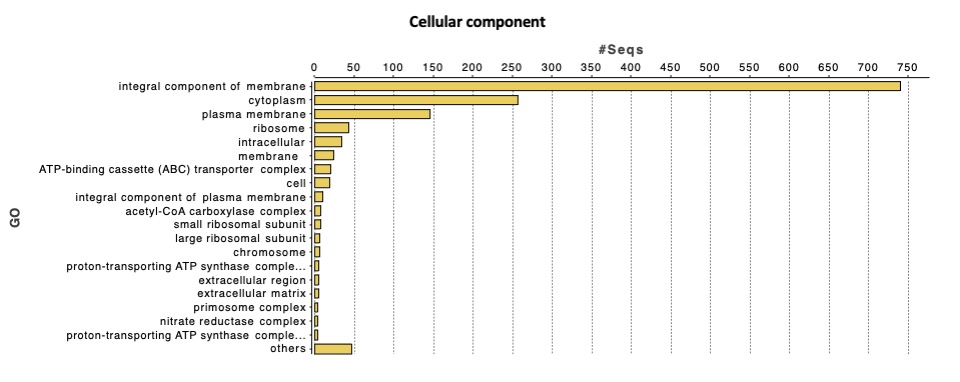

Supplement: Supplementary file 1 [file Data_Sheet_1.zip › Figure S1b.JPEG]

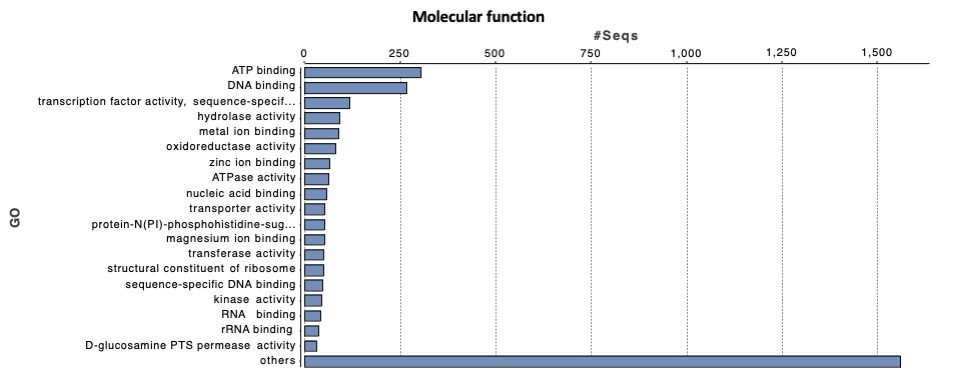

Supplement: Supplementary file 1 [file Data_Sheet_1.zip › Figure S1c.JPEG]

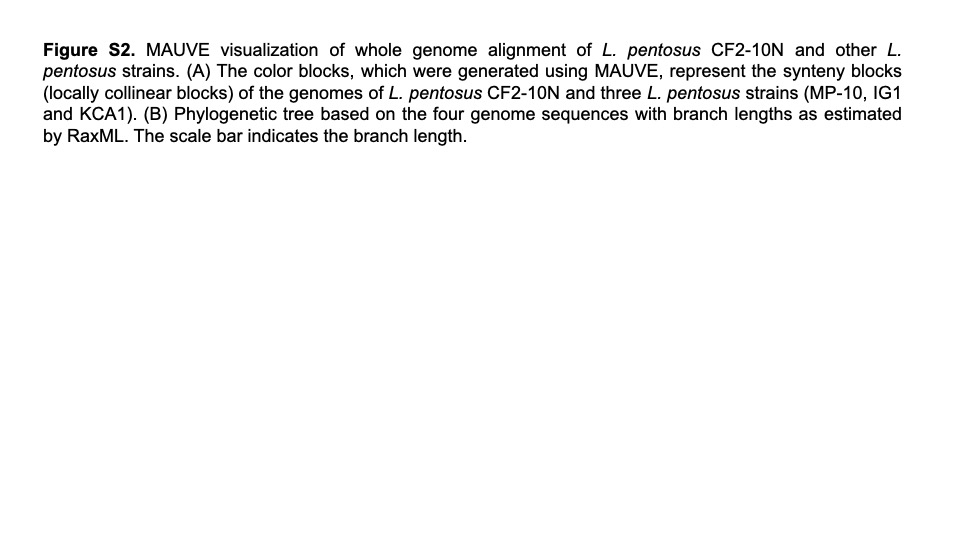

Supplement: Supplementary file 1 [file Data_Sheet_1.zip › Figure S2 caption.JPEG]

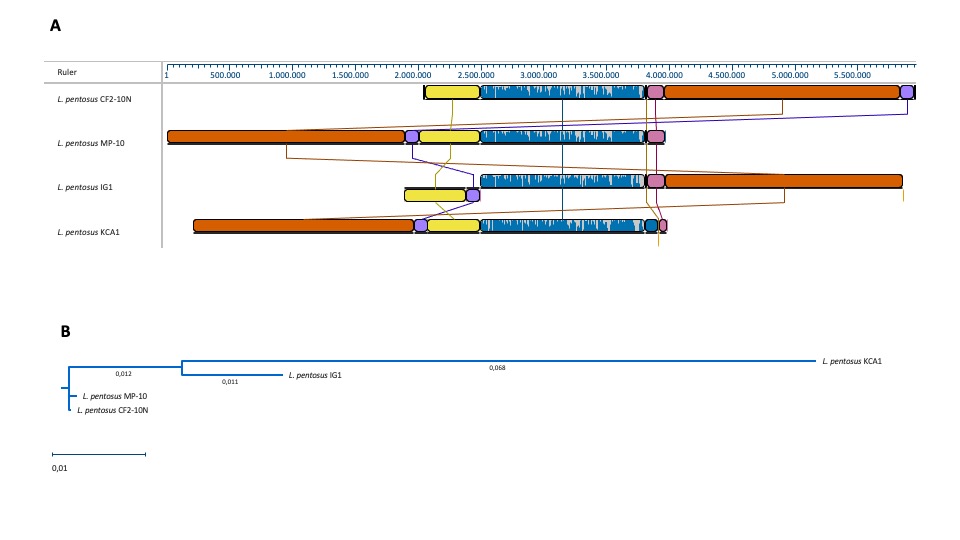

Supplement: Supplementary file 1 [file Data_Sheet_1.zip › Figure S2.JPEG]

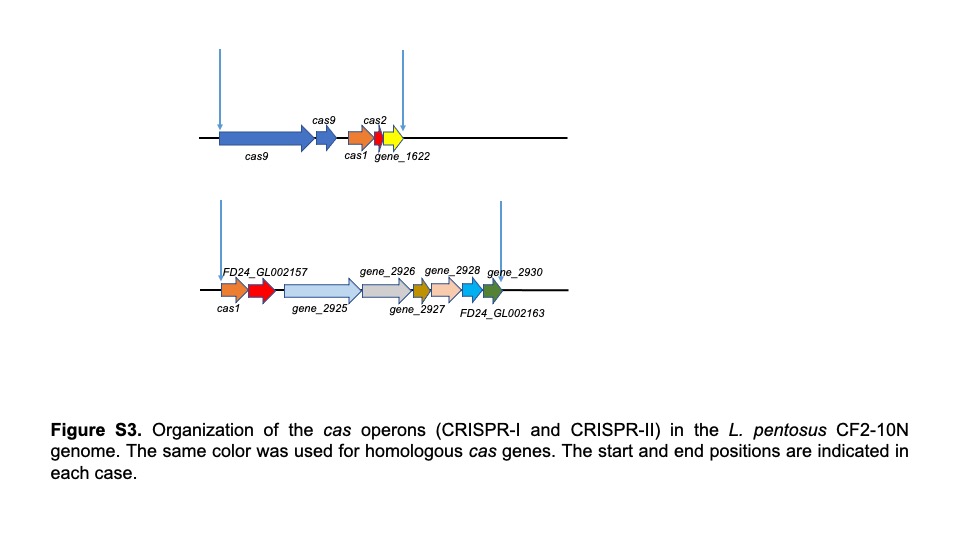

Supplement: Supplementary file 1 [file Data_Sheet_1.zip › Figure S3.JPEG]
